# Genome-wide transposon mutagenesis of *Proteus mirabilis*: Essential genes, fitness factors for catheter-associated urinary tract infection, and the impact of polymicrobial infection on fitness requirements

**DOI:** 10.1371/journal.ppat.1006434

**Published:** 2017-06-14

**Authors:** Chelsie E. Armbruster, Valerie Forsyth-DeOrnellas, Alexandra O. Johnson, Sara N. Smith, Lili Zhao, Weisheng Wu, Harry L. T. Mobley

**Affiliations:** 1 Department of Microbiology and Immunology, University of Michigan Medical School, Ann Arbor, Michigan, United States of America; 2 Department of Microbiology and Immunology, Jacobs School of Medicine and Biomedical Sciences, State University of New York at Buffalo, Buffalo, New York, United States of America; 3 Department of Biostatistics, University of Michigan School of Public Health, Ann Arbor, Michigan, United States of America; 4 Department of Computational Medicine & Bioinformatics, University of Michigan Medical School, Ann Arbor, Michigan, United States of America; University of California Davis School of Medicine, UNITED STATES

## Abstract

The Gram-negative bacterium *Proteus mirabilis* is a leading cause of catheter-associated urinary tract infections (CAUTIs), which are often polymicrobial. Numerous prior studies have uncovered virulence factors for *P*. *mirabilis* pathogenicity in a murine model of ascending UTI, but little is known concerning pathogenesis during CAUTI or polymicrobial infection. In this study, we utilized five pools of 10,000 transposon mutants each and transposon insertion-site sequencing (Tn-Seq) to identify the full arsenal of *P*. *mirabilis* HI4320 fitness factors for single-species *versus* polymicrobial CAUTI with *Providencia stuartii* BE2467. 436 genes in the input pools lacked transposon insertions and were therefore concluded to be essential for *P*. *mirabilis* growth in rich medium. 629 genes were identified as *P*. *mirabilis* fitness factors during single-species CAUTI. Tn-Seq from coinfection with *P*. *stuartii* revealed 217/629 (35%) of the same genes as identified by single-species Tn-Seq, and 1353 additional factors that specifically contribute to colonization during coinfection. Mutants were constructed in eight genes of interest to validate the initial screen: 7/8 (88%) mutants exhibited the expected phenotypes for single-species CAUTI, and 3/3 (100%) validated the expected phenotypes for polymicrobial CAUTI. This approach provided validation of numerous previously described *P*. *mirabilis* fitness determinants from an ascending model of UTI, the discovery of novel fitness determinants specifically for CAUTI, and a stringent assessment of how polymicrobial infection influences fitness requirements. For instance, we describe a requirement for branched-chain amino acid biosynthesis by *P*. *mirabilis* during coinfection due to high-affinity import of leucine by *P*. *stuartii*. Further investigation of genes and pathways that provide a competitive advantage during both single-species and polymicrobial CAUTI will likely provide robust targets for therapeutic intervention to reduce *P*. *mirabilis* CAUTI incidence and severity.

## Author summary

*Proteus mirabilis* is a common cause of single-species and polymicrobial catheter-associated urinary tract infections (CAUTIs). Prior studies have uncovered *P*. *mirabilis* virulence factors for single-species ascending UTI, but little is known concerning pathogenesis during CAUTI or polymicrobial infection. Using transposon insertion-site sequencing (Tn-Seq), we performed a global assessment of *P*. *mirabilis* fitness factors for CAUTI while simultaneously determining how coinfection with another CAUTI pathogen, *Providencia stuartii*, alters *P*. *mirabilis* fitness requirements. This approach provides six important contributions to the field: 1) the first global estimation of *P*. *mirabilis* genes essential for growth, 2) validation of a role for known *P*. *mirabilis* fitness factors during CAUTI, 3) identification of novel fitness factors, 4) identification of core fitness factors for both single-species and polymicrobial CAUTI, 5) identification of single-species fitness factors that are complemented during polymicrobial infection, and 6) identification of factors that only provide a competitive advantage during polymicrobial infection. We further demonstrate that the CAUTI model can be used to examine the interplay between fitness requirements of both species during coinfection. Investigation of fitness requirements for other pathogens during single-species and polymicrobial CAUTI will elucidate complex interactions that contribute to disease severity and uncover conserved targets for therapeutic intervention.

## Introduction

The Gram-negative bacterium *Proteus mirabilis* thrives in a wide variety of environments, including soil, water sources, sewage, and as a commensal in the intestinal tract of humans and animals [1, 2]. *P*. *mirabilis* has also been identified as the causative agent of numerous human illnesses including cystitis, pyelonephritis, prostatitis, as well as intra-abdominal, wound, eye, and burn infections [2]. While it is capable of causing uncomplicated urinary tract infections (UTIs), this organism is a much more common cause of catheter-associated UTI (CAUTI) [3–5]. Indeed, we recently identified *P*. *mirabilis* as the most common cause of CAUTIs in twelve Michigan nursing homes [6]. CAUTIs are frequently polymicrobial [7, 8], and *P*. *mirabilis* is one of the most common organisms present during polymicrobial urine colonization and infection [3, 6]. UTIs and CAUTIs involving *P*. *mirabilis* are typically complicated by the formation of bladder and kidney stones (urolithiasis) and permanent renal damage [9–11], and may progress to bacteremia [12, 13]. Despite these potentially severe complications of *P*. *mirabilis* infection, there are no currently licensed vaccines available for this organism and multidrug-resistant isolates are increasingly common [14, 15].

No previous studies have explored *P*. *mirabilis* genes essential for growth *in vitro*, but numerous fitness and virulence factors have been examined in a murine model of ascending UTI, including but not limited to urease, fimbriae, and hemolysin (see [2, 16] for review). Fitness factors for colonization of the urinary tract have also been explored in three non-saturating signature-tagged mutagenesis (STM) studies in the ascending UTI model using *P*. *mirabilis* strain HI4320, which achieved a combined 70% theoretical coverage of the genome and revealed traditional virulence factors, metabolic pathways important for infection, and fitness factors with no prior links to *P*. *mirabilis* pathogenicity [17–19]. For *P*. *mirabilis* infection studies, the murine model of ascending UTI is generally considered to represent complicated UTI due to the occurrence of urolithiasis in this infection model [20]. However, we recently adapted a murine model of CAUTI for investigation of *P*. *mirabilis* pathogenicity, and verified that maintenance of a catheter segment within the bladder dramatically increases inflammation and infection severity [21]. Thus, different genes and pathways are likely required for fitness in the catheterized bladder environment than those identified in the ascending UTI model.

An additional consideration in identifying fitness and virulence factors of *P*. *mirabilis* is the frequent involvement of this organism in polymicrobial infections. We recently determined that other uropathogens, such as *Escherichia coli*, *Enterococcus* species, and *Providencia stuartii*, are capable of influencing *P*. *mirabilis* urease activity [21]. Co-culture with *P*. *stuartii* also enhanced *P*. *mirabilis* cytotoxicity independent of urease activity, altered the immune response to infection, and resulted in greater tissue damage, indicating that other virulence factors are affected by polymicrobial interactions [21]. All prior studies of *P*. *mirabilis* fitness factors have been conducted with pure cultures of *P*. *mirabilis* in isolation, yet the potential clearly exists for other organisms to influence expression of *P*. *mirabilis* virulence factors, metabolic requirements, and factors required for adaptation to changes in the bladder environment in response to the presence of a second pathogen. In this study, we generated a genome-saturating library of transposon mutants and utilized transposon insertion-site sequencing (Tn-Seq) to identify *P*. *mirabilis* genes essential for growth in rich medium and the full arsenal of fitness factors for single-species infection in a CAUTI model, while concurrently determining how fitness requirements changed during polymicrobial infection with *P*. *stuartii*, a common co-colonizing partner of *P*. *mirabilis* [3, 4, 22–25].

## Results

### Generation of five transposon mutant libraries in *Proteus mirabilis* HI4320

Based on the *P*. *mirabilis* HI4320 genome size (4.063 Mbp, with approximately 3747 genes [26]), it is estimated that 34,249 transposon mutants are required for 99.99% probability of full genome coverage [27]. A similar strategy as detailed by Crimmins et al. [28] for maximum colonization density was used to determine the appropriate transposon library pool density. From our recent investigation of experimental CAUTI in CBA/J mice, the minimum colonization density achieved by *P*. *mirabilis* in the urine, bladder, and kidneys of mice 4-days post inoculation is expected to be ~1x10^4^ CFU [21]. Preliminary experiments confirmed a minimum bladder colonization of 1x10^4^ CFU/gram of tissue at 4-h and 4-days post-inoculation, and indicated lack of a significant bottleneck in the CAUTI model (S1 Fig). We therefore concluded that generation of transposon pools containing 1x10^4^ transposon mutants would be ideal. Approximately 50,000 transposon mutants from three independent matings were collected and pooled in groups of 1x10^4^ mutants to generate five transposon mutant libraries. Randomness of insertions was verified by Southern blot, and the majority of mutants harbored only one transposon insertion as expected (S2 Fig).

### Identification of transposon insertion-sites and estimation of genes essential for growth in rich medium

**Fig 1:**
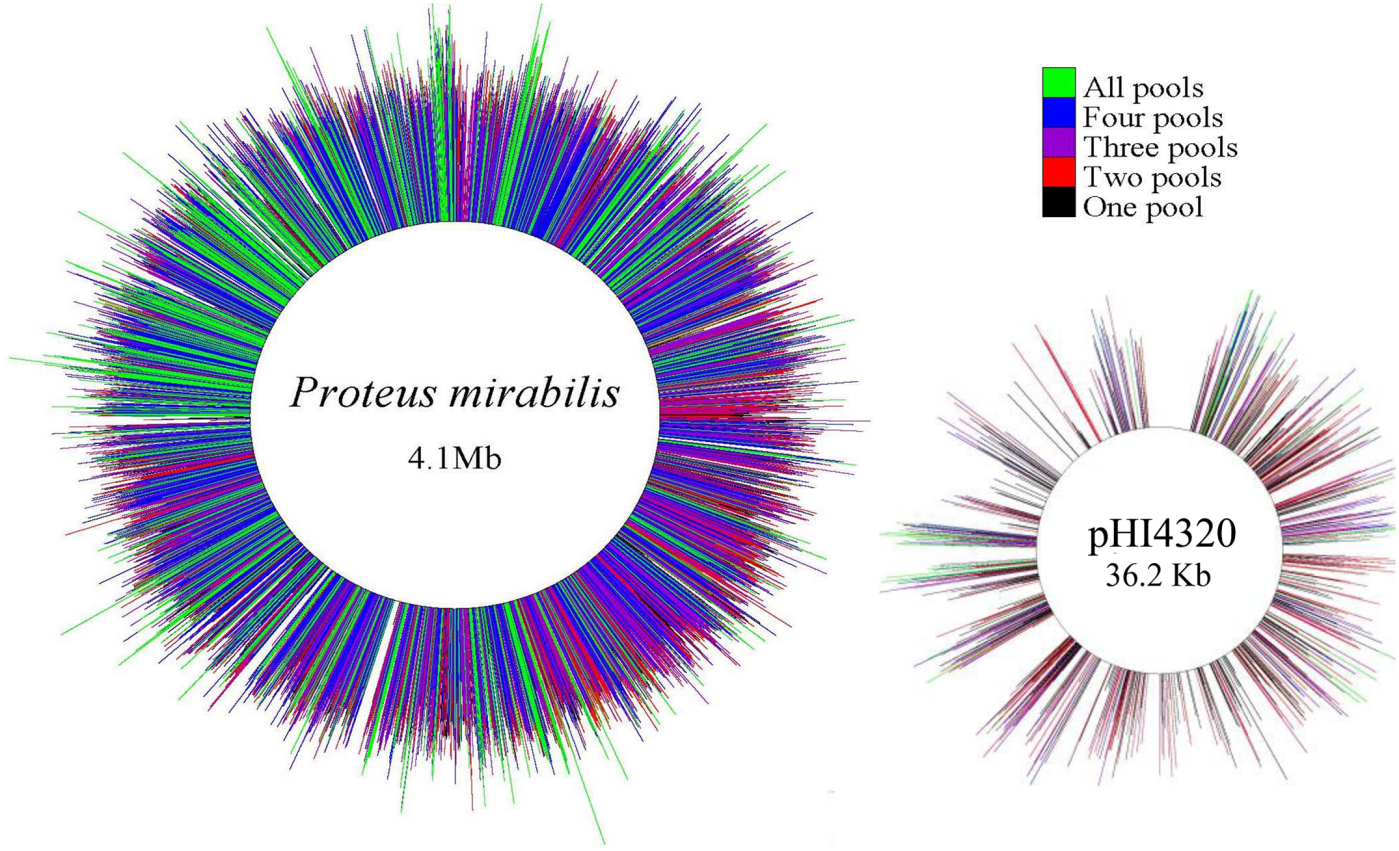
Saturation of the *Proteus mirabilis* HI4320 genome and plasmid pHI4320 with transposon insertions. Chromosomal and plasmid maps (not to scale) indicating the location of all transposon insertions contained in all five of the input pools. On average, the *P*. *mirabilis* transposon pools contained an insertion every 50.9 bp, with ~21,694 open reading frame insertions per pool. Regions without insertions represent estimated essential genes and intergenic regions. Each line represents a single insertion site from the input samples, and the length of the line represents the log_10_ number of reads recovered from each insertion site. The color of the line indicates the number of pools in which each insertion site was identified: all five pools (green), four pools (blue), three pools (purple), two pools (red), one pool (black).

**Fig 2:**
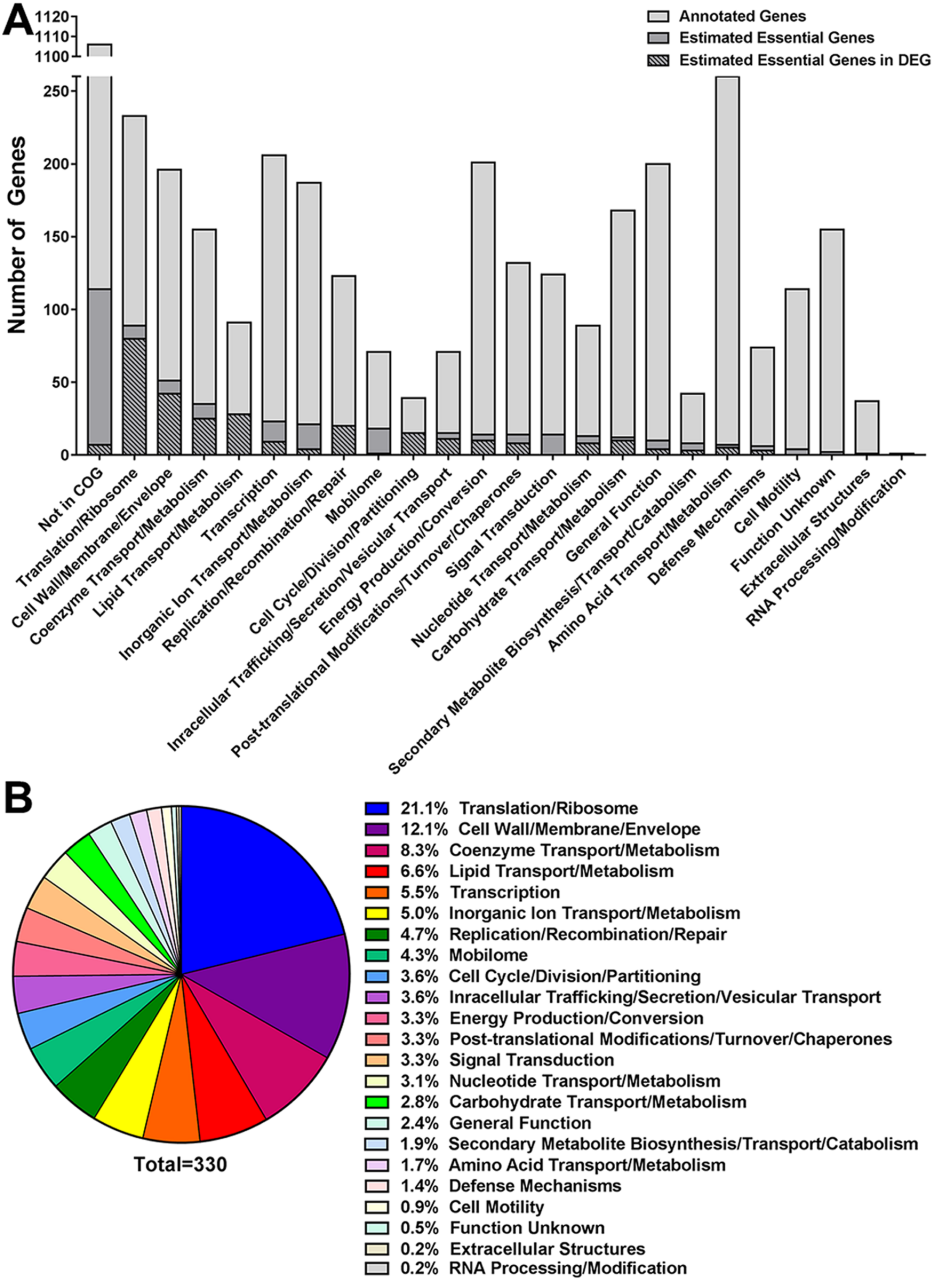
Functional categories of *P*. *mirabilis* HI4320 estimated essential genes. A Bayesian mixture model was used to identify genes estimated to be essential for *P*. *mirabilis* growth in LB medium based on absence or underrepresentation of transposon insertions in these genes from all five input pools (see Materials and methods). (A) The number of annotated genes encoded by HI4320 pertaining to each Cluster of Orthologous Groups of proteins (COG) is indicated by the light gray bars. Within each COG, the genes estimated to be essential for growth in LB broth are indicated by the dark gray bars, and the number of these estimated essential genes present in the Database of Essential Genes (DEG) is indicated by crosshatching. (B) The percentage of estimated essential genes belonging to each COG are displayed in descending order.

Raw Illumina reads were filtered and transposon insertion-sites were uniquely mapped to genomic coordinates using a method adapted from the previously published work by Goodman et al. [29]. Saturation was achieved for the *P*. *mirabilis* HI4320 chromosome and 36 Kb plasmid (pHI4320), and the majority of insertion-sites were represented in three or more input pools (Fig 1). However, there were a few noticeable gaps in both the chromosome and plasmid maps. To determine if these gaps represented genes essential for growth of *P*. *mirabilis* in LB medium, a Bayesian mixture model was used to estimate essentiality based on absence or underrepresentation of transposon insertions in these genes within the input pools (see Materials and methods). The model identified 436 genes (11.6% of the 3747 genes encoded by *P*. *mirabilis* HI4320) as potentially essential for *P*. *mirabilis* growth (S1 Table), 279 (64.0%) of which were present in the bacterial section of the Database of Essential Genes (DEG, http://www.essentialgene.org/). Three hundred thirty-three of the estimated essential genes had a Cluster of Orthologous Groups of proteins (COG) assignment (Fig 2).

Among the list of *P*. *mirabilis* estimated essential genes are numerous genes listed as essential in the majority of bacterial species present in DEG, including genes pertaining to cell cycle control and division (such as the *fts* cell division proteins), cell wall biogenesis, replication (including DNA polymerase I, DNA polymerase III, and DNA gyrase), RNA polymerase σ^70^ (*rpoD*), σ^32^ (*rpoH*), and σ^24^ (*rpoE*), nucleoid protein H-NS (*hns*), numerous ribosomal proteins and tRNA synthetases, and ATP synthase. There are five ATP-dependent proteases in bacteria that comprise the majority of energy-dependent protein degradation: HflB (FtsH), the Clp proteases (ClpAP, ClpXP, and ClpYQ), and the Lon protease [30]. Insertions in three of these proteases (HflB, ClpAP, and ClpXP) were absent from the input pools and therefore identified as essential for growth of *P*. *mirabilis* in rich medium, underscoring the importance of degrading misfolded and unstable proteins for *P*. *mirabilis* growth in rich medium. The replication initiation protein PMIP01 on the *P*. *mirabilis* plasmid (pHI4320) was also identified as essential.

### Primary screen of *P*. *mirabilis* transposon mutant libraries in a murine model of CAUTI

**Fig 3:**
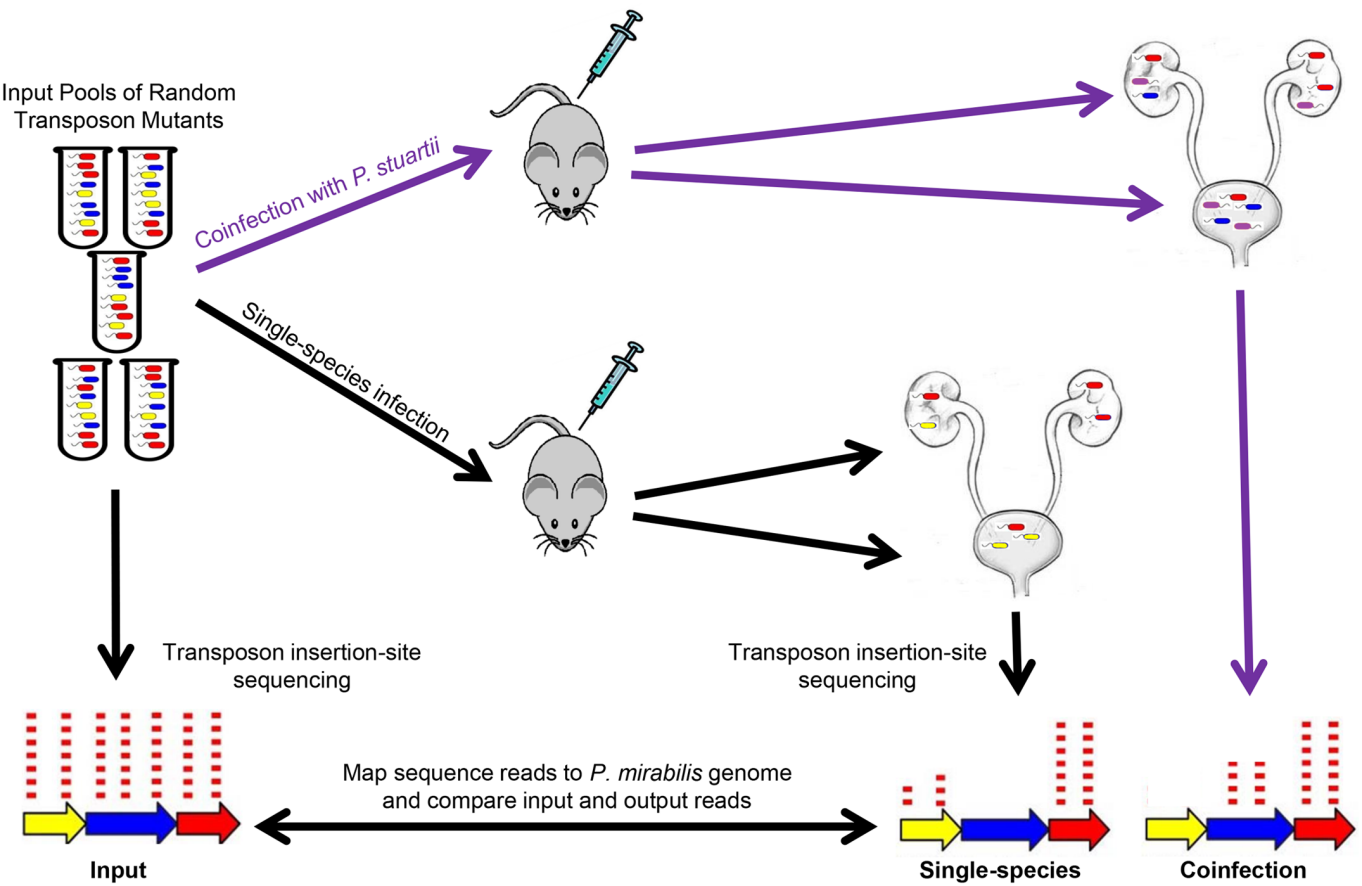
Conceptual model of single-species and polymicrobial CAUTI Tn-Seq. For each of five transposon mutant library pools, mice were infected as follows: 1) 5–10 CBA/J mice were transurethrally inoculated with 1x10^5^ CFU of the transposon library for single-species infection, and 2) 5–10 CBA/J mice were inoculated with 1x10^5^ CFU of a 1:1 mixture of the transposon library and wild-type *P*. *stuartii* BE2467 (purple) for coinfection. Thus, for each input pool, the single-species infections and coinfections were conducted in parallel to utilize the same input inoculum. Input and output samples were enriched for transposon-containing sequences and subjected to next generation Illumina sequencing of the transposon-chromosome junctions. The resulting reads were mapped to the *P*. *mirabilis* genome, and the abundance of reads at each insertion site from all output samples were compared to the input samples to determine a fold change for each gene. The gene in yellow represents a candidate *P*. *mirabilis* fitness factor for single-species CAUTI that is even more important during coinfection; the gene in blue represents a *P*. *mirabilis* fitness factor for single-species CAUTI that is no longer important during coinfection; the gene in red represents a factor that does not contribute to *P*. *mirabilis* CAUTI and was therefore recovered at a similar density from the infection output pools as the input pools.

Infection studies to screen for *P*. *mirabilis* fitness factors during single-species and polymicrobial CAUTI were conducted in parallel to utilize the same inocula as the input samples for each infection type. A conceptual model of the infection and sequencing scheme is shown in Fig 3. For each of the five transposon library pools, 5–10 CBA/J mice were transurethrally inoculated with 1x10^5^ CFU (10X coverage of each mutant within the pool), and 5–10 CBA/J mice were inoculated with 1x10^5^ CFU of a 1:1 mixture of the transposon library and wild-type *P*. *stuartii* BE2467 (5x10^4^ CFU of the *P*. *mirabilis* transposon mutant library for 5X coverage of each mutant within the pool). In all cases, a 4 mm segment of sterile silicone catheter tubing was carefully advanced into the bladder during inoculation and retained for the duration of the study as described previously [21]. Due to the level of encrustation in mice infected with urease-positive organisms, the catheter segments were generally embedded in the bladder tissue and therefore were not removed prior to homogenization of bladder samples. Thus, all CFUs recovered from bladder samples actually represent colonization of the catheter segment as well as the bladder.

**Fig 4:**
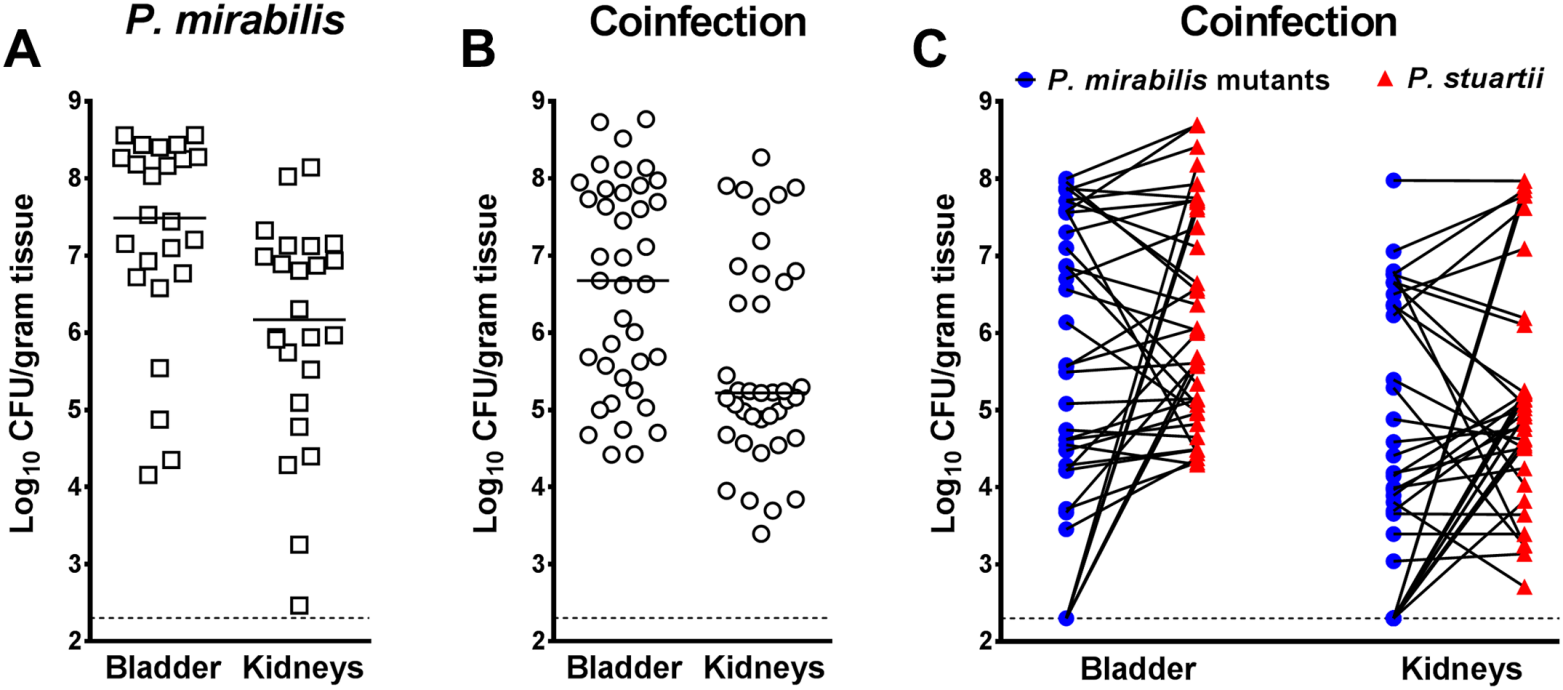
Colonization by *P*. *mirabilis* transposon mutants during single-species and polymicrobial CAUTI. For each transposon mutant library pool, 5–10 CBA/J mice were transurethrally inoculated with 1x10^5^ CFU of the transposon library for single-species infection (A), and 5–10 CBA/J mice were inoculated with 1x10^5^ CFU of a 1:1 mixture of the transposon library and wild-type *P*. *stuartii* BE2467 for coinfection (B and C). In all cases, a 4 mm segment of catheter tubing was retained in the bladder for the duration of the study. Mice were sacrificed 4 days post-inoculation, and the bladder and kidneys were homogenized, and an aliquot was plated onto LB agar to determine bacterial burden. The remaining homogenate was fully plated for isolation of bacterial genomic DNA and sequencing. (A and B) Each symbol represents total CFU/gram of tissue from an individual mouse during single-species infection (A) or coinfection (B), and error bars indicate the median. (C) The majority of coinfected mice were highly colonized by both bacterial species. Blue circles represent *P*. *mirabilis* CFU/gram of tissue and red triangles represent *P*. *stuartii* CFU/gram of tissue, with values from a single mouse connected by a black line. Dashed lines indicate limit of detection.

Single-species infections and coinfections with the *P*. *mirabilis* transposon mutant pools resulted in comparable bladder and kidney colonization in all mice (Fig 4A and 4B). Differential plating revealed that the majority of coinfected mice were highly colonized by both *P*. *mirabilis* and *P*. *stuartii*, as expected (Fig 4C). In cases where an individual mouse exhibited low colonization by *P*. *mirabilis* in either the bladder or kidneys (<1x10^4^ CFU), the samples from that mouse were excluded from further study. Bladder and kidney output samples for sequencing were chosen from the most highly colonized mice as follows: four mice from the single-species infection group and four from the coinfection group, per transposon pool, resulting in 20 bladder samples and 20 kidney samples from each infection type. A fitness index was calculated for each gene by taking into account the number of unique insertion-sites within that gene and the depth of reads at each insertion-site for the combined output samples recovered from the organs of infected mice compared to the combined input pools (see Materials and methods).

### Identification of candidate *P*. *mirabilis* fitness factors for single-species CAUTI

Tn-Seq from single-species CAUTI identified 629 genes (16.8% of the total genes encoded by *P*. *mirabilis* HI4320) as candidate fitness factors. Due to the stringent cutoffs that were used for estimation of fitness factors (see Materials and methods section), the range of fitness defects was compressed, spanning a 2- to 40-fold reduction in recovery from output samples compared to the input samples. The top candidate fitness factors for colonization of the catheterized bladder (excluding tRNAs, pseudogenes, and genes not present in COG) are shown in Table 1. Four of these genes were previously identified by signature-tagged mutagenesis (STM) in the ascending UTI model [18, 19], and eight were previously shown to be upregulated by *P*. *mirabilis* in the ascending UTI model [31]. Importantly, many of the top hits for bladder colonization represent multiple genes in an operon, including the phosphate ABC transporter (*pstSCAB*) and the cytochrome *bo*3 quinol oxidase (*cyoABCDE*). Top candidate fitness factors for kidney colonization (again excluding tRNAs, pseudogenes, and genes not present in COG) are shown in Table 2. These include the same four genes identified by STM that were important for bladder colonization, and fourteen genes that were upregulated by *P*. *mirabilis* in the ascending UTI model [31].

**Table 1:** Top *P*. *mirabilis* fitness factors for colonization of the catheterized bladder.

PMI Number	Gene	Name	Fold Change
PMI0818		hypothetical protein, putative HicA toxin	27.86
PMI0817		phage protein, putative HicB antitoxin	23.13
PMI2893	pstS*	phosphate ABC transporter substrate-binding protein	21.33
PMI2894	pstC*	phosphate ABC transporter membrane protein 1	17.00
PMI0106	*cyoC*	cytochrome bo3 quinol oxidase subunit 3	16.62
PMI1676	*cspC*	cold-shock DNA-binding protein family	16.59
PMI1210	^	transcriptional regulator, PadR family	16.52
PMI3496		HTH-type transcriptional regulator / antitoxin HigA	16.43
PMI2895	*pstA*	phosphate ABC transporter membrane protein 2	15.28
PMI3251	*tufB*	elongation factor Tu	15.12
PMI0105	*cyoE*^	protoheme IX farnesyltransferase	15.01
PMI1861	*iscR*	transcriptional regulator, BadM/Rrf2 family	14.98
PMI2047	*pdhR*^	transcriptional regulator, GntR family	14.66
PMI3622	*fis*	DNA-binding protein Fis	14.40
PMI2087	*leuD*	3-isopropylmalate dehydratase, small subunit	13.76
PMI0105A	*cyoD*	cytochrome bo3 quinol oxidase subunit 4	13.59
PMI0117	*lon*	ATP-dependent proteinase/serine peptidase	12.41
PMI0550		antitoxin ParD1/3/4	12.22
PMI0108	*cyoA*^	cytochrome bo3 quinol oxidase subunit 2	11.80
PMI3377	*rplI*	LSU ribosomal protein L9P	11.69
PMI2879	^	plasmid-related protein	11.25
PMI0107	*cyoB*^	cytochrome bo3 quinol oxidase subunit 1 apoprotein	11.21
PMI2046	aceE*^	pyruvate dehydrogenase E1 component	9.81
PMI2930	*glpD*	homodimeric glycerol 3-phosphate dehydrogenase (quinone)	9.79
PMI1376	*pspA*	phage shock protein A (PspA) family protein	9.71
PMI3623	dusB*	tRNA-U20-dihydrouridine synthase	9.52
PMI2771	*hupA*	bacterial nucleoid protein HU alpha subunit	9.48
PMI0536	*ucaA*	major fimbrial subunit	8.37
PMI2416	*deoC*^	deoxyribose-phosphate aldolase	8.11
PMIP34		plasmid segregation oscillating ATPase ParF	7.85
PMI1734	*nrdA*	ribonucleoside-diphosphate reductase class Ia alpha subunit	7.46
PMI0140		hypothetical protein	7.33
PMI1401		MFS-family transporter	7.16
PMI0765	*ompF*	outer membrane porin	7.15
PMI1859	*iscU*	FeS assembly scaffold apoprotein IscU	7.09
PMI3399	*argR*	transcriptional regulator, ArgR family	6.94

*Denotes genes previously identified as fitness factors for ascending UTI by STM

^Denotes genes previously determined to be upregulated during ascending UTI

**Table 2:** Top *P*. *mirabilis* fitness factors for kidney colonization.

PMI Number	Gene	Name	Fold Change
PMI0818		hypothetical protein, putative HicA toxin	18.77
PMI2893	pstS*	phosphate ABC transporter substrate-binding protein	13.85
PMI0105A	*cyoD*	cytochrome bo3 quinol oxidase subunit 4	13.52
PMI1376	*pspA*	phage shock protein A (PspA) family protein	13.10
PMI3251	*tufB*	elongation factor Tu	12.74
PMI2047	*pdhR*^	transcriptional regulator, GntR family	12.02
PMI0105	*cyoE*^	protoheme IX farnesyltransferase	11.41
PMI2894	pstC*	phosphate ABC transporter membrane protein 1	11.37
PMI0550		antitoxin ParD1/3/4	10.97
PMI0117	*lon*	ATP-dependent proteinase/serine peptidase	10.26
PMI2895	*pstA*	phosphate ABC transporter membrane protein 2	9.80
PMI2046	aceE*^	pyruvate dehydrogenase E1 component	9.63
PMI1203	*fnr*	fumarate and nitrate reduction regulatory protein	9.54
PMI1828	*ptsH*^	PTS system phosphocarrier protein	9.02
PMI3622	*fis*	DNA-binding protein Fis	8.94
PMI2896	*pstB*	phosphate ABC transporter ATP-binding protein	8.62
PMI2879	^	plasmid-related protein	8.44
PMI1734	*nrdA*	ribonucleoside-diphosphate reductase class Ia alpha subunit	8.35
PMI3699		hypothetical protein	8.24
PMI3623	dusB*	tRNA-U20-dihydrouridine synthase	8.16
PMI0118	*hupB*	DNA-binding protein HU-beta	7.98
PMI2141	*agaR*^	transcriptional regulator, DeoR family	7.62
PMI1829	*ptsI*^	phosphoenolpyruvate—protein phosphotransferase	7.60
PMI1210	^	transcriptional regulator, PadR family	7.52
PMI0107	*cyoB*^	cytochrome bo3 quinol oxidase subunit 1 apoprotein	7.34
PMI0106	*cyoC*	cytochrome bo3 quinol oxidase subunit 3	7.23
PMI0543	*fur*	ferric uptake regulation protein	7.06
PMI3252	*bfd*^	bacterioferritin-associated ferredoxin	6.97
PMI2930	*glpD*	homodimeric glycerol 3-phosphate dehydrogenase (quinone)	6.74
PMI1676	*cspC*	cold-shock DNA-binding protein family	6.71
PMI2414	*deoB*^	phosphopentomutase	6.65
PMI2416	*deoC*^	deoxyribose-phosphate aldolase	6.54
PMI1401		MFS-family transporter	6.38
PMI0108	*cyoA*^	cytochrome bo3 quinol oxidase subunit 2	6.37
PMI3054	*mioC*	flavoprotein involved in biotin synthesis	6.30
PMI0140		hypothetical protein	6.11
PMI3216	*hslV*^	HslV component of HslUV peptidase/threonine peptidase	6.01
PMI1409	*lpp*	major outer membrane lipoprotein (murein-lipoprotein)	5.97
PMI2045	*aceF*^	acetyltransferase component of pyruvate dehydrogenase	5.96

*Denotes genes previously identified as fitness factors for ascending UTI by STM

^Denotes genes previously determined to be upregulated during ascending UTI

**Fig 5:**
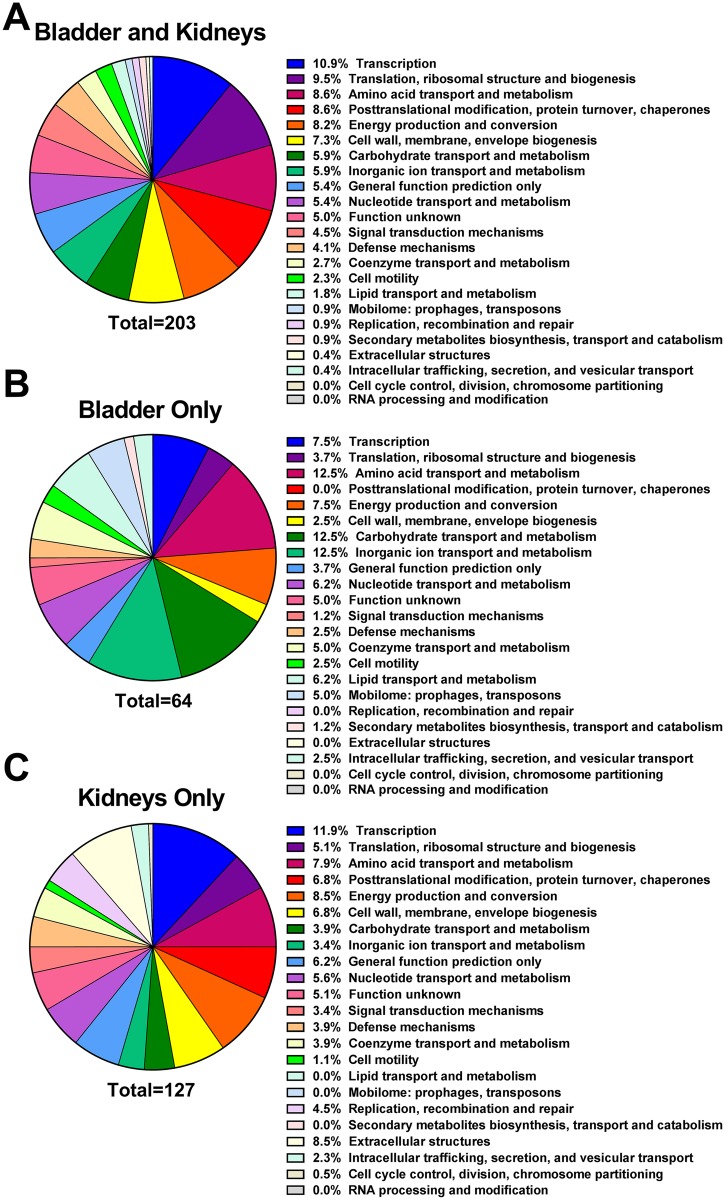
Functional categories of *P*. *mirabilis* fitness factors for single-species CAUTI. (A) The percentage of 203 candidate *P*. *mirabilis* fitness factors required for colonization of both the catheterized bladder and kidneys during single-species CAUTI belonging to each COG are displayed in descending order. (B) COG categories represented by 64 candidate fitness factors for bladder colonization that did not contribute to kidney colonization. (C) COG categories represented by 127 candidate fitness factors for kidney colonization that did not contribute to bladder colonization.

Upon further analysis, fitness factors for single-species CAUTI fell into three categories: 93 genes for colonization of the catheterized bladder that were not significant for kidney colonization (S2 Table), 209 genes for kidney colonization that were not significant for bladder colonization (S3 Table), and 286 genes that were important for colonization of both organs (S4 Table). Fitness factors for colonization of each organ were randomly distributed across the chromosome and plasmid (S3 Fig). Of the 286 genes important for both bladder and kidney colonization, 203 were present in COG: 24 pertained to transcription (10.9% of functional category assignments); 21 (9.5%) translation, ribosomal structure, and biogenesis; 19 (8.6%) amino acid transport and metabolism; 19 (8.6%) post-translational modification, protein turnover, and chaperones; 18 (8.2%) energy production and conversion; and 16 (7.3%) cell wall and envelope biogenesis (Fig 5A). Regarding fitness factors for bladder colonization alone, 64 of the 93 candidate factors were present in COG and revealed a greater proportion of genes pertaining to amino acid transport, carbohydrate transport and metabolism, and inorganic ion transport and metabolism than the genes important for colonization of both organs, and fewer candidate genes pertaining to translation and cell wall biogenesis (Fig 5B). Interestingly, the candidate fitness factors for kidney colonization alone followed a similar distribution as the genes important for colonization of both the bladder and kidneys with a few notable exceptions, including fewer genes pertaining to translation or carbohydrate transport and metabolism, and more pertaining to replication, recombination, and repair and extracellular structures. (Fig 5C). Thus, functional categories important for kidney colonization are likely to be involved in fitness during bladder colonization as well.

Overall, of the fifty-four *P*. *mirabilis* fitness factors previously identified by signature-tagged mutagenesis (STM) in the ascending UTI model [17–19], 31 (57%) were significant for CAUTI (denoted with a “*” in the supplemental tables). These include the phosphate ABC transporter (*pstSCAB*), pyruvate dehydrogenase (*aceE* and *aceF*), an AsnC transcriptional regulator (PMI1431), a d-methionine ABC transporter protein (*metN*), inosine-5’-monophosphate dehydrogenase (*guaB*), a bifunctional polymyxin resistance protein (*arnA*), a polysaccharide deacetylase next to the Arn operon (PMI1046), a dihydrouridine synthase (*dusB*), and a hypothetical protein (PMI3700). Urease is another well-known fitness factor for *P*. *mirabilis* UTI and prior STM studies that we previously verified as important for colonization in the CAUTI model [21], and a urease accessory protein (*ureG*) required for the catalytically-active enzyme was also identified in the present CAUTI Tn-Seq.

#### Validation of candidate fitness factors for single-species CAUTI

To validate the primary screen, mutations were made in eight genes to assess four general categories of fitness defects: 1) factors critical for both bladder and kidney colonization (>5-fold change for both organs), 2) factors that to contribute to both bladder and kidney colonization (2-4-fold change for both organs), 3) factors important for colonization of the kidneys but not the bladder, and 4) factors that were not estimated to significantly contribute to fitness in the CAUTI model. Three mutants pertaining to Category 1 were generated for validation: the Lon ATP-dependent protease (*lon*, ~12-fold defect in bladder and kidney colonization), an arginine repressor (*argR*, ~6-fold defect), and the ClpYU ATP-dependent protease (*hslU*, *~*5-fold defect). Three mutants pertaining to Category 2 were used for validation: glutamine synthetase (*glnA*, ~3-fold defect in bladder and kidney colonization), the bifunctional polymyxin resistance protein previously identified by STM in the ascending UTI model (*arnA*, ~3-fold defect), and phospholipase A (*pldA*, *~*2-fold defect). A single mutant pertaining to Category 3, the putative high-affinity nickel export protein (PMI1518, 2.2-fold defect in kidney colonization), was generated for validation. Similarly, Category 4 was represented by a single mutant of *ilvD*, an enzyme involved in branched chain amino acid (BCAA) biosynthesis, as insertions in this gene were recovered at approximately the same ratio from both organs during single-species infection as from the input samples.

**Fig 6:**
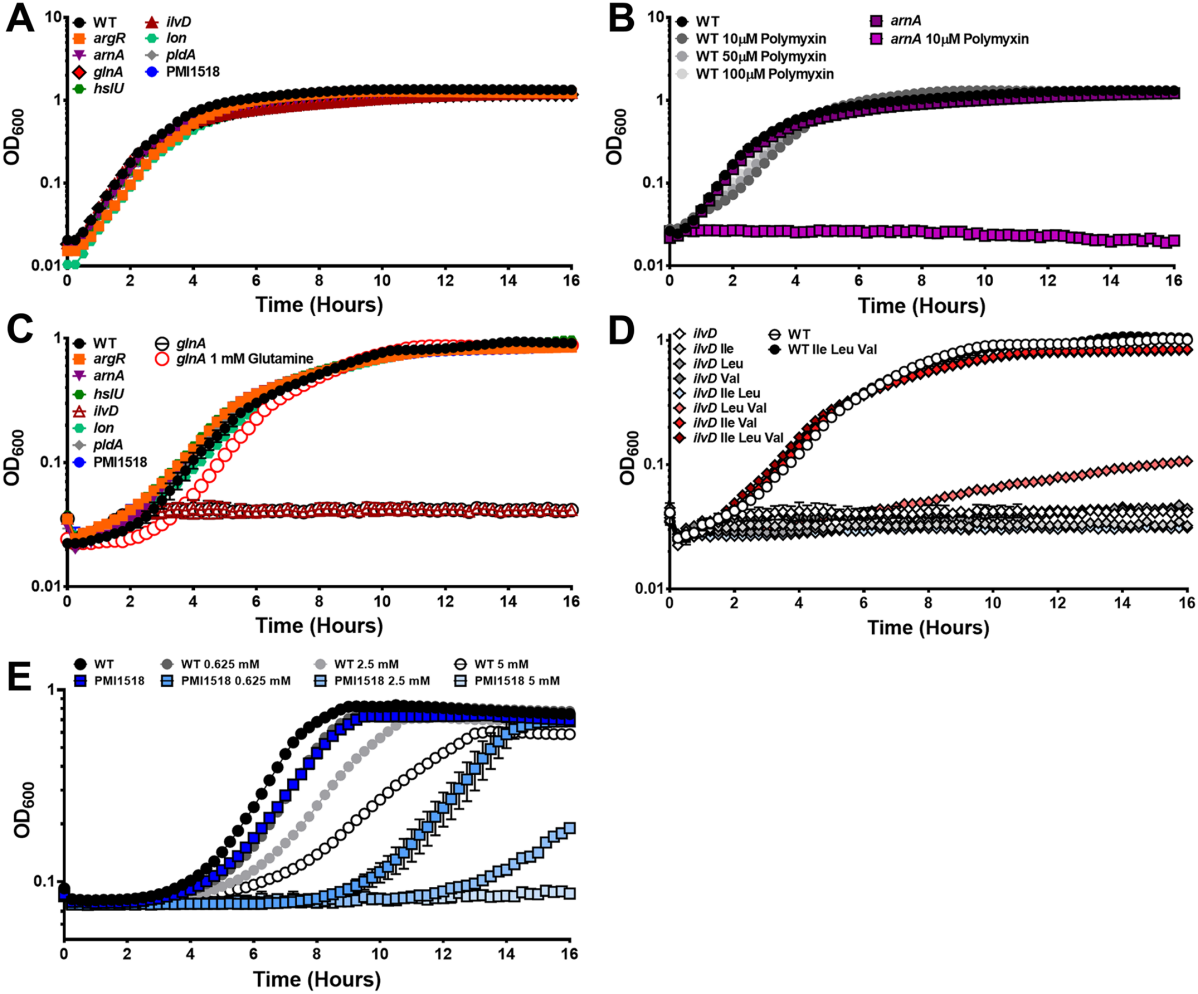
Growth of wild-type *P*. *mirabilis* and mutants in LB broth and minimal medium. Growth of *P*. *mirabilis* HI4320 and mutants was measured in LB broth (A and B) and PMSM minimal medium (C, D, and E) with the following supplements: (A) none, (B) polymyxin B, (C) 1 mM l-glutamine, (D) 10 mM total of the listed BCAAs, and (E) nickel sulfate. Graphs are representative of at least three independent experiments. Error bars represent mean ± standard deviation (SD) from at least five technical replicates. Differences in growth between strains and treatment conditions were determined to be significant by two-way ANOVA.

None of the mutants exhibited growth defects in LB broth (Fig 6A). Since ArnA mediates resistance to polymyxin B and other cationic antimicrobial peptides, we tested susceptibility of wild-type *P*. *mirabilis* and the *arnA* mutant to polymxyin B (Fig 6B). As expected, the *arnA* mutant was significantly more susceptible than wild-type, exhibiting growth inhibition at 10 μM polymyxin B, while wild-type *P*. *mirabilis* retained normal growth characteristics in 100 μM polymyxin B. When cultured in minimal medium, the *glnA* and *ilvD* mutants were auxotrophic for l-glutamine and BCAAs (isoleucine, leucine, and valine), respectively, and none of the other mutants exhibited growth defects (Fig 6C). Growth of the *glnA* mutant was complemented by the addition of l-glutamine (Fig 6C), as previously reported [32]. Growth of the *ilvD* mutant was partially restored by supplementation with leucine and valine, and fully complemented by supplementation with isoleucine and valine or all three BCAAs (Fig 6D).

Nickel is an essential cofactor for numerous enzymes including urease. However, none of the genes encoding the putative Nik ABC transporter (PMI2943-PMI2947) were significant fitness factors for *P*. *mirabilis* during single-species CAUTI. While the ability to scavenge and import metal is a critical feature of numerous pathogens, transition metal efflux is also important to avoid toxicity [33]. For instance, copper mobilization is a host response to UTI and uropathogenic *Escherichia coli* employs multiple copper efflux systems for protection against copper toxicity [33–35]. The high-affinity nickel efflux protein encoded by PMI1518 was a significant fitness factor for kidney colonization. We therefore hypothesized that this transporter may function to control nickel homeostasis and protect *P*. *mirabilis* from nickel toxicity. Growth of wild-type *P*. *mirabilis* in minimal medium was perturbed by the addition of nickel sulfate, although not completely inhibited (Fig 6E). The PMI1518 mutant was significantly more sensitive to nickel than wild-type, displaying a dramatic decrease in growth with the addition of 0.625 mM nickel sulfate and complete growth inhibition at 5 mM nickel sulfate (Fig 6E). Thus, PMI1518 encodes a nickel exporter that contributes to nickel tolerance.

**Fig 7:**
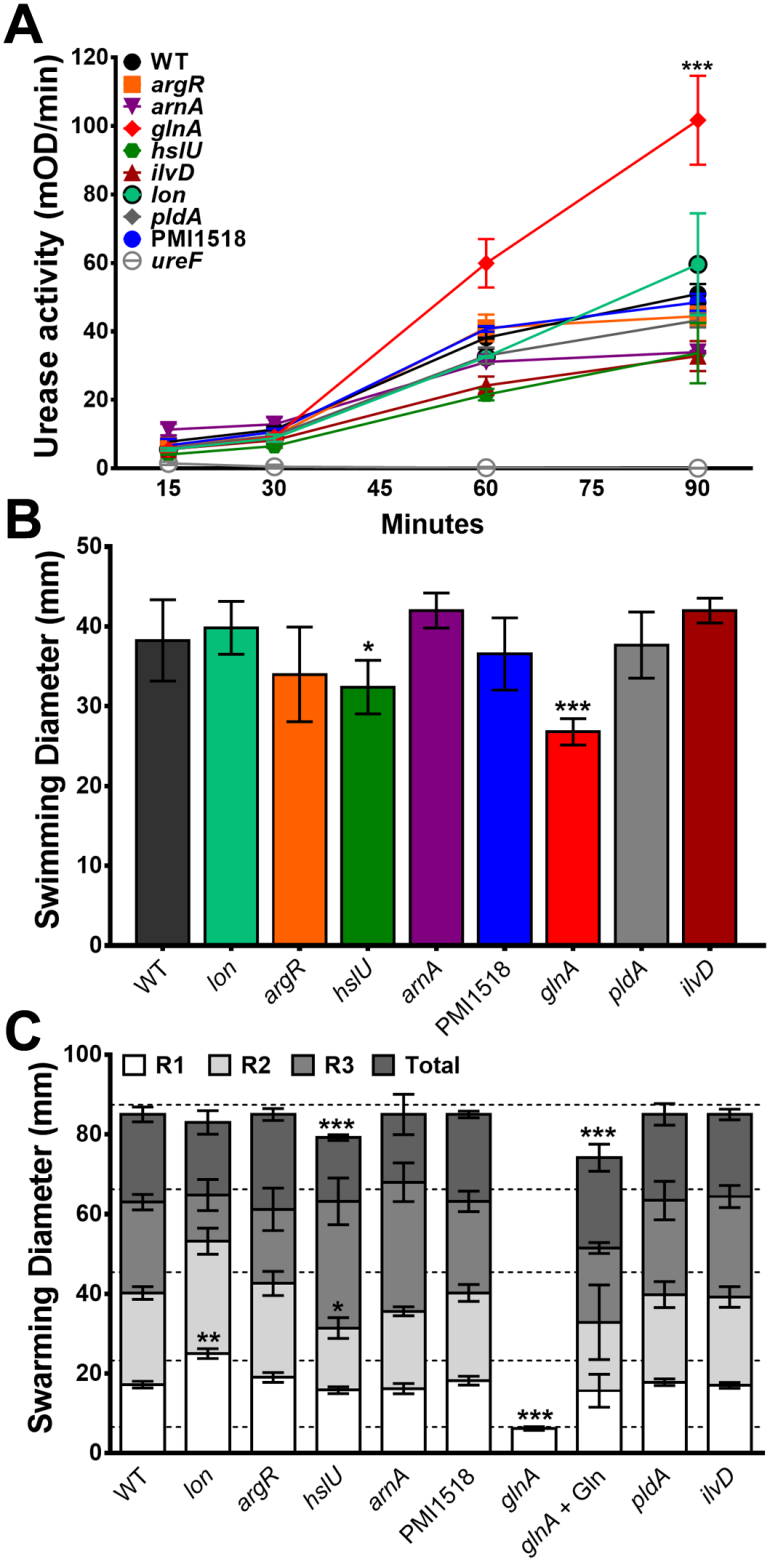
Urease activity and motility of wild-type *P*. *mirabilis* and mutants. (A) *P*. *mirabilis* and mutants were cultured in filter-sterilized human urine to measure urease activity, expressed as the mean change in optical density per minute (mOD/min) of the indicator dye phenol red during a 5-minute kinetic read at each indicated timepoint. Graph is representative of at least three independent experiments. Error bars represent mean ± SD from at least three technical replicates. ***P<0.001 by two-way ANOVA. (B) Swimming motility diameter in Mot agar, compiled from three independent experiments with three technical replicates each. Error bars represent mean ± SD. *P<0.05, ***P<0.001 compared to wild-type by Student’s *t*-test. (C) Diameter of the 1^st^ (R1), 2^nd^ (R2), and 3^rd^ (R3) swarm rings to the consolidation zone and total swarm diameter for *P*. *mirabilis* and mutants compiled from three independent experiments with at least two technical replicates each. Dashed lines indicate average swarm ring diameter for wild-type *P*. *mirabilis*. Error bars represent mean ± SD. *P<0.05, **P<0.01, and ***P<0.001 compared to wild-type *P*. *mirabilis* by two-way ANOVA with post-hoc test for significance.

With potential growth defects explored, we next sought to determine the impact of each mutation on two key aspects of *P*. *mirabilis* virulence: urease activity and motility. None of the mutants exhibited a defect in urease activity (Fig 7A), although the *glnA* mutant had an apparent increase; however, this may be due to accumulation of ammonia from loss of glutamine synthetase rather than an actual increase in enzyme activity. All mutants were capable of swimming in semi-solid motility agar, although the *glnA* and *hslU* mutants exhibited slightly decreased swimming diameter (Fig 7B). Similarly, all mutants were capable of swarming on permissive medium except the *glnA* mutant (Fig 7C), which was fully complemented by the addition of concentrations of l-glutamine present in human urine, as previously reported [32]. The *hslU* mutant exhibited a slight decrease in the diameter of the second swarm ring and the total distance migrated, while the *lon* mutant exhibited a hyper-swarming phenotype with increased diameter of the first swarm ring.

**Fig 8:**
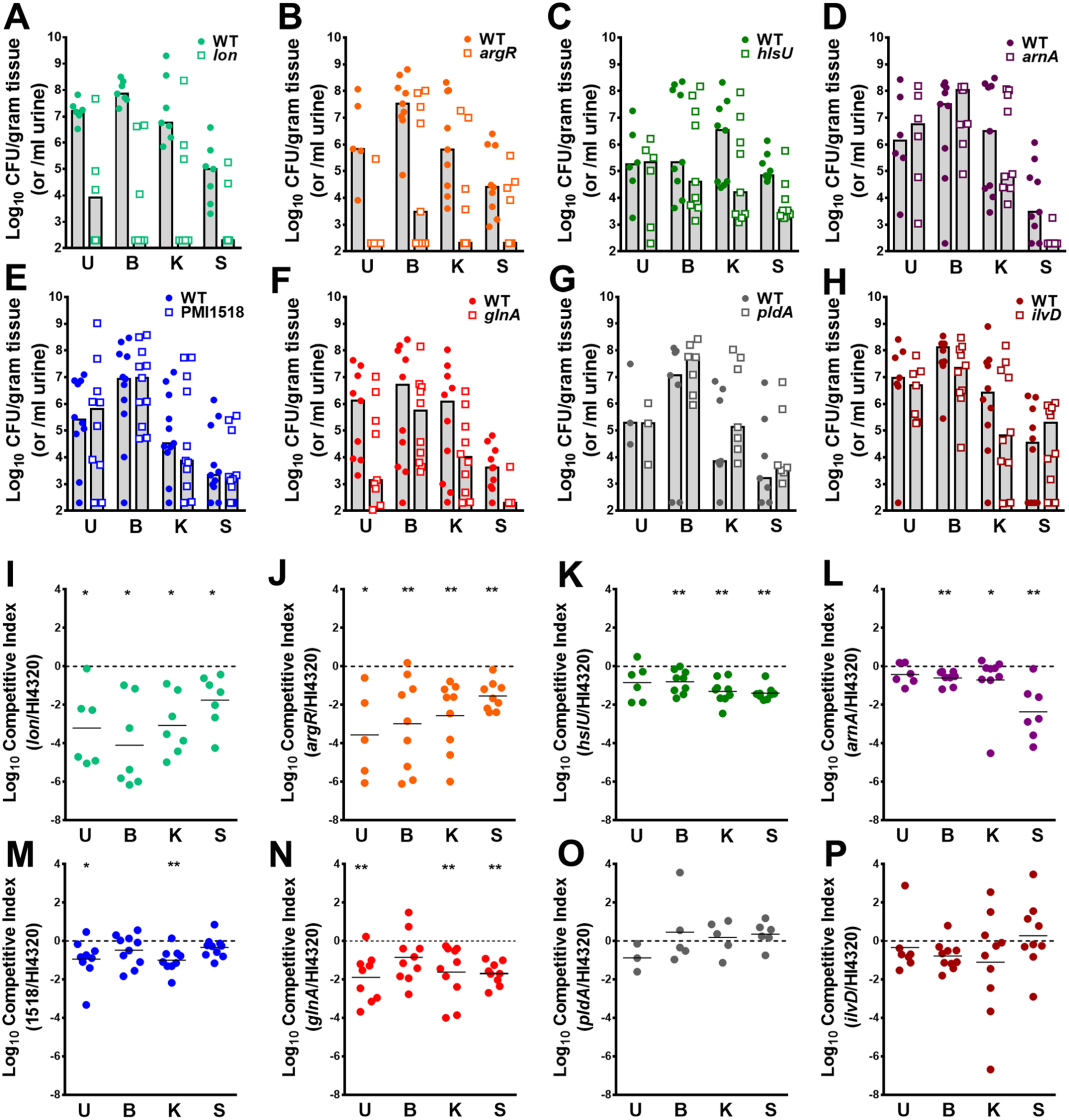
Validation of candidate *P*. *mirabilis* fitness factors for single-species CAUTI. CBA/J mice were transurethrally inoculated with 1x10^5^ CFU of a 1:1 mixture of wild-type *P*. *mirabilis* and an isogenic mutant. In all cases, a 4 mm segment of catheter tubing was retained in the bladder for the duration of the study. Urine was collected and mice were sacrificed 4 days post-inoculation, and the catheterized bladder, kidneys, and spleen were homogenized and plated onto LB agar with and without kanamycin to determine bacterial burden of wild-type *P*. *mirabilis* and the mutant (A-H). Each data point represents the Log_10_ CFUs recovered from an individual mouse, and gray bars represent the median. A competitive index was calculated for each mutant on a per-mouse basis, for organs in which the mutant and wild-type were above the limit of detection, using the ratio of mutant to wild-type in each organ divided by the ratio of mutant to wild-type from the inoculum (I-P, see Materials and methods). (A and I) *lon*, (B and J) *argR*, (C and K) *hslU*, (D and L) *arnA*, (E and M) PMI1518, (F and N) *glnA*, (G and O) *pldA*, and (H and P) *ilvD*. Solid lines represent the median. Dashed lines indicate a competitive index of 1, or a 1:1 ratio of mutant to wild-type. *P<0.05 and **P<0.01 by the Wilcoxon signed rank test.

To test fitness of these mutants during direct co-challenge with the parental *P*. *mirabilis* strain, CBA/J mice were transurethrally inoculated with 1x10^5^ CFU of a 1:1 mixture of the mutant and wild-type *P*. *mirabilis*. Urine was collected when possible for determination of CFUs, as well as the catheterized bladder, kidneys, and spleen (Fig 8A–8H). A competitive index was calculated on a per-mouse basis for all organs in which the mutant and wild-type *P*. *mirabilis* were recovered above the limit of detection (Fig 8I–8P). Five mutants (*lon*, *argR*, *hlsU*, *arnA*, and PMI1518) exhibited the same colonization defects as observed in the primary screen. The *glnA* mutant was outcompeted by the wild-type strain in the urine and kidneys, but only trended towards being outcompeted in the bladder (Fig 8N). The *pldA* mutant did not exhibit a defect in any organ, and was therefore considered to be a false-positive (Fig 8O). Notably, the *ilvD* mutant, which served as a negative control, was not significantly outcompeted by the wild-type strain in any organ, confirming that BCAA synthesis is not required for *P*. *mirabilis* fitness during single-species CAUTI (Fig 8P). It is also worth noting that the *lon*, *argR*, *hslU*, *arnA*, and *glnA* mutants were outcompeted by wild-type in the spleen, indicating a potential role for these gene products in urosepsis. Thus, single-species CAUTI Tn-Seq fitness defects were validated in 7/8 (88%) mutants.

**Fig 9:**
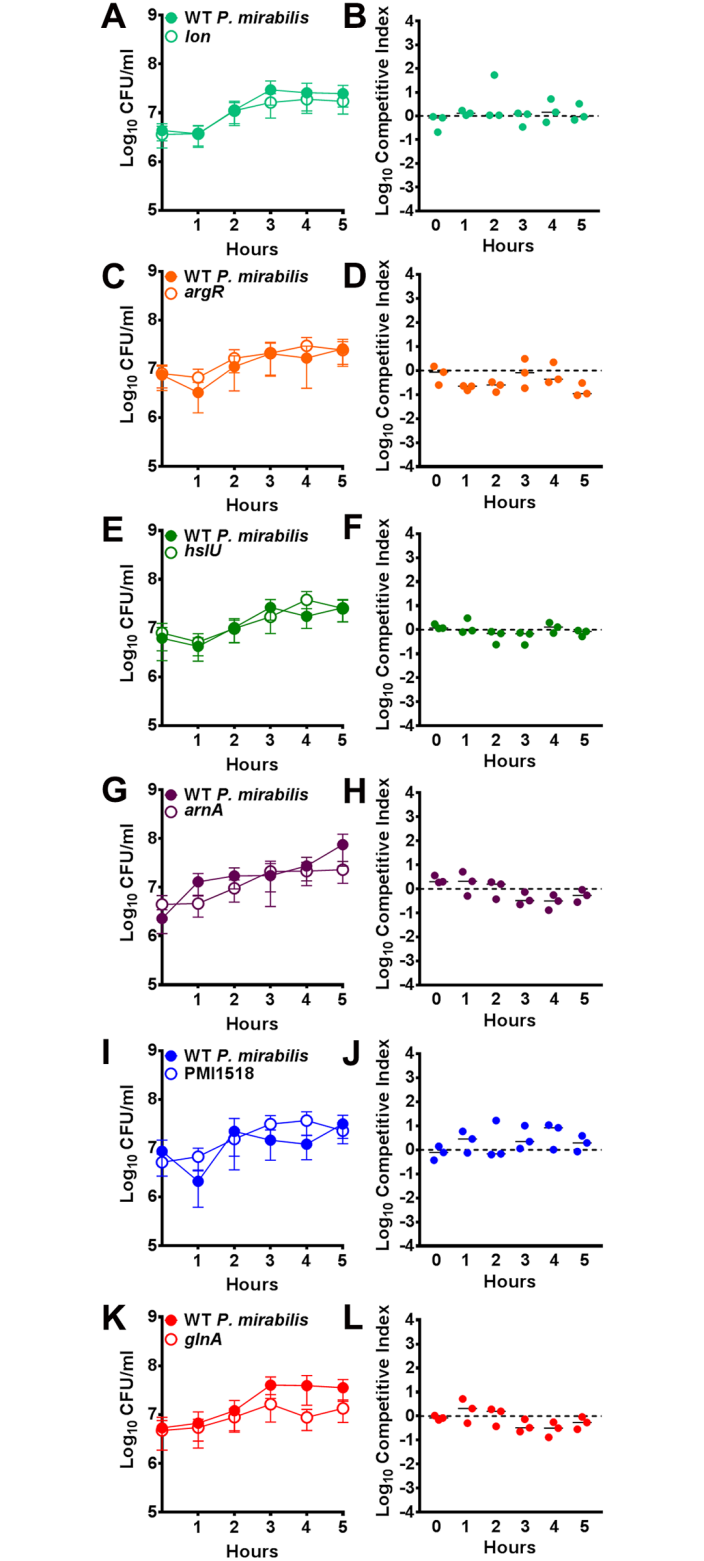
*In vitro* co-challenge of *P*. *mirabilis* mutants in human urine. Filter-sterilized pooled human urine from healthy donors was inoculated with a 1:1 mixture of wild-type *P*. *mirabilis* and the following mutants that exhibited significant fitness defects *in vivo*: (A and B) *lon*, (C and D) *argR*, (E and F) *hslU*, (G and H) *arnA*, (I and J) PMI1518, and (K and L) *glnA*. Cultures were incubated at 37°C for 5 hours, and sampled hourly for determination of CFUs (A, C, E, G, I, and K). Error bars represent mean and error for three independent replicates. No differences in growth between mutants and wild-type were detected by two-way ANOVA with post-hoc test for significance. A competitive index was calculated for each mutant at each hourly timepoint using the ratio of mutant to wild-type at the time of inoculation (B, D, F, H, J, and L). None of the mutants exhibited a significant fitness defect or advantage during growth in urine by the Wilxocon signed rank test.

To determine if the observed fitness defects were *in vivo* specific, co-challenge experiments were repeated *in vitro* using pooled urine from healthy human donors (Fig 9). All of the mutants were capable of growing to similar levels as wild-type within human urine, and none exhibited a significant fitness defect during co-culture *in vitro*. The fitness defects exhibited by these mutants are therefore specifically related to the host environment of the catheterized urinary tract and cannot be replicated in a test tube.

### Identification of candidate *P*. *mirabilis* fitness factors for polymicrobial CAUTI

Tn-Seq from coinfection with *P*. *stuartii* revealed 1570 candidate *P*. *mirabilis* fitness factors, ranging from 2- to 30-fold reduction of transposon insertions in these genes from the combined output samples compared to the input samples. Candidate fitness factors represented 217/629 (34.5%) of the genes identified above for single-species CAUTI, indicating substantial overlap between the two infection models, as well as 1353 additional factors that may specifically contribute to colonization during coinfection. Sixty-two genes were candidate fitness factors for bladder colonization alone (S5 Table), 10 of which were also important for bladder colonization during single-species infection. These bladder-specific fitness factors, regardless of infection type, include two members of a carbohydrate ABC transporter (*ugpC* and *ugpE*), a nucleoid-associated protein (*ndpA*), the anti σ^E^ protein (*rseA*), an HxlR-family transcriptional regulator, and a siderophore receptor (*ireA*).

213 genes were candidate fitness factors for kidney colonization alone (S6 Table), 61 of which were also important for kidney colonization during single-species infection. The majority of the kidney-specific fitness factors, regardless of infection type, pertained to amino acid transport and metabolism (including the d-serine ammonia-lyase *dsdA* and the l-serine ammonia lyase *sdaA*), cell wall and envelope biogenesis, energy production and conversion (including succinate dehydrogenase and formate acetyltransferase), posttranslational modification and protein turnover (*sufD*, an iron-regulated ABC transporter permease protein, and *surA*, a periplasmic chaperone for outer membrane proteins), transcriptional regulation (including the *phoP* two-component response regulator, and an RpiR-family transcriptional regulator encoded by PMI2974), and four plasmid-encoded genes (PMIP09, which encodes the *pilX4* conjugal transfer protein, PMIP10, PMIP28, and PMIP30). The Sec-independent protein translocase TatC (twin arginine transporter) was also identified as important for kidney colonization but not bladder colonization in both infection types.

**Fig 10:**
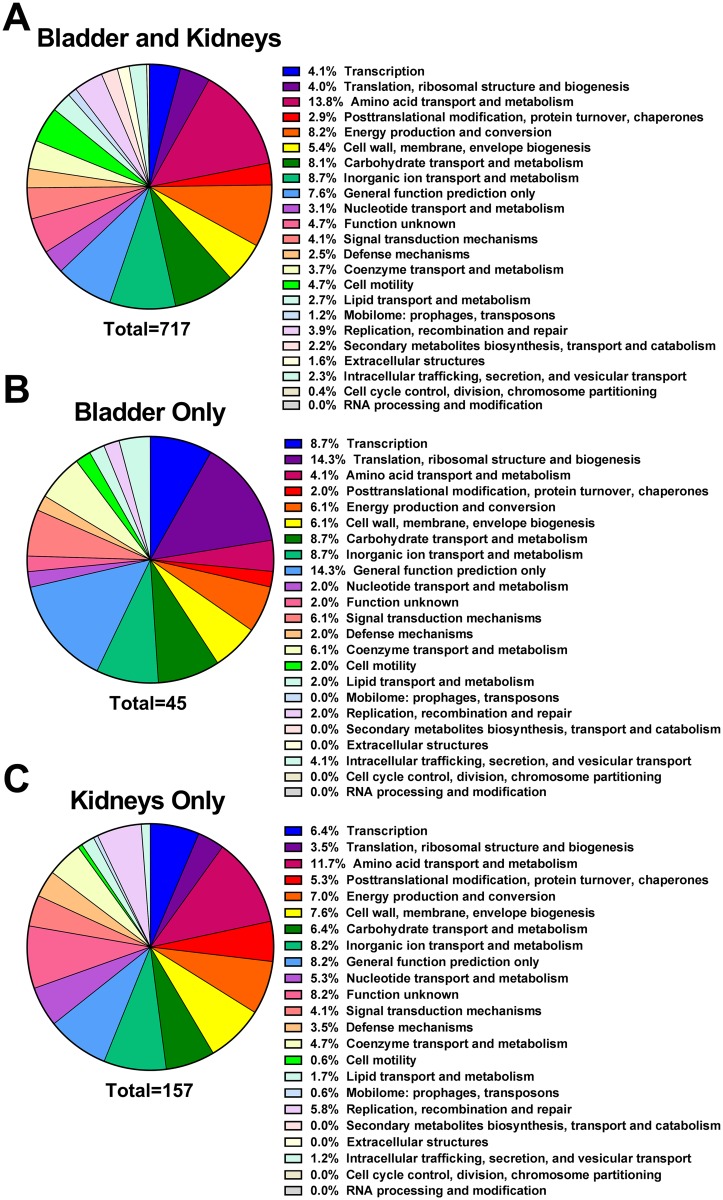
Functional categories of *P*. *mirabilis* fitness factors for polymicrobial CAUTI. (A) The percentage of 717 candidate *P*. *mirabilis* fitness factors required for both bladder and kidney colonization during single-species CAUTI belonging to each COG are displayed in same the order as in Fig 5 for comparison. (B) COG categories of 45 candidate fitness factors for bladder colonization that did not contribute to kidney colonization. (C) COG categories of 157 candidate fitness factors for kidney colonization that did not contribute to bladder colonization.

909 genes were important for both bladder and kidney colonization during coinfection (S7 Table), 717 of which were present in COG and primarily pertained to transport and metabolism of amino acids, inorganic ions, carbohydrates, as well as energy production and conversion (Fig 10A). The COG functional categories represented by *P*. *mirabilis* fitness factors during coinfection followed a similar trend as single-species infection: fitness factors for bladder colonization alone revealed a greater proportion of genes pertaining to translation and transcription compared to the genes important for colonization of both organs (Fig 10B), and fitness factors for kidney colonization alone followed a similar distribution as genes important for colonization of the bladder and kidneys, albeit with an increase in factors with unknown function and a decrease in factors pertaining to cell motility (Fig 10C).

Fifteen of the genes important for colonization of both the bladder and kidneys during coinfection were also important for colonization of both organs during single-species infection. Seven of these genes have unknown functions or are not in COG; the other genes pertain to translation, ribosomal structure, and biogenesis (*dusC*, PMI3283, and *rimI*, an alanine acetyltransferase), cell wall/envelop biogenesis and defense (*mdtA*, a multidrug resistance efflux transporter), motility (PMI2642), and inorganic ion transport and metabolism (*ppaA*). The factors encoded by these genes appear to represent a putative core set of fitness requirements for *P*. *mirabilis* colonization of the murine urinary tract.

Intriguingly, there were also 109 genes for which transposon insertions resulted in a fitness defect during single-species infection but provided a competitive advantage during polymicrobial CAUTI (S8 Table). Seventy-nine of these genes were present in COG, and included factors involved in energy production and conversion, transcription, carbohydrate transport and metabolism, inorganic ion transport (particularly iron and phosphate), amino acid transport and metabolism, and nucleotide transport and metabolism.

#### Validation of candidate fitness factors for polymicrobial CAUTI

Three candidate fitness factors were chosen for validation of the coinfection Tn-Seq results: the high-affinity nickel transport protein (PMI1518, 2.2-fold defect in single-species kidney colonization, 5.1–6.7-fold defect in bladder and kidney colonization, respectively, during coinfection), the dihydroxy-acid dehydratase involved in BCAA biosynthesis (*ilvD*, no defect for single-species CAUTI, 2.2–3.0-fold defect in bladder and kidney colonization during coinfection), and the Lon ATP-dependent protease (12.3–12.4-fold defect for single-species bladder and kidney colonization, 2.7–6.8-fold advantage during coinfection). Importantly, the fitness defects identified during single-species CAUTI by Tn-Seq were validated for all three mutants during direct co-challenge with the parental *P*. *mirabilis* strain, as shown in Fig 8.

**Fig 11:**
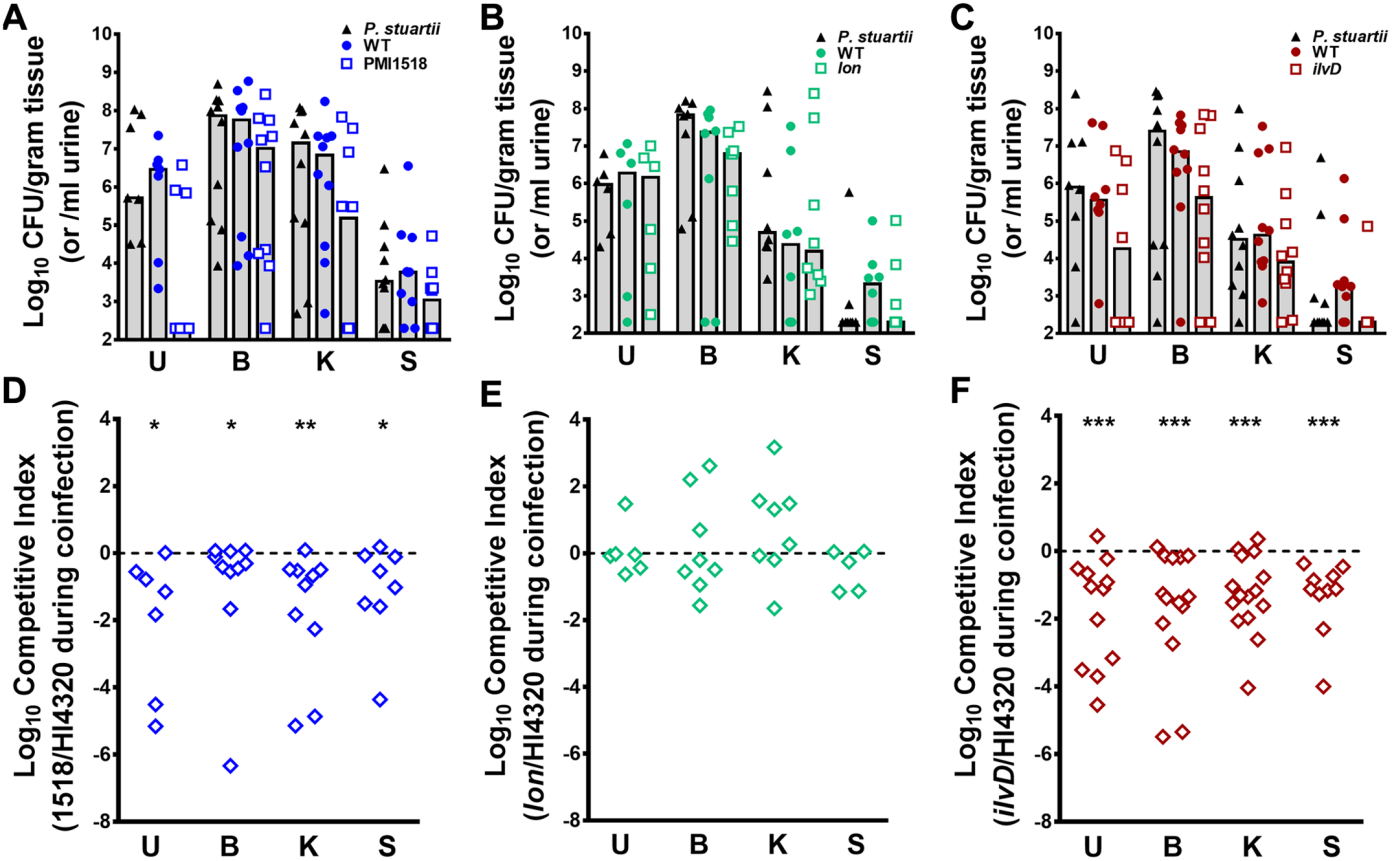
Validation of candidate *P*. *mirabilis* fitness factors for polymicrobial CAUTI. CBA/J mice were transurethrally inoculated with 1x10^5^ CFU of the following mixture: 5x10^4^ CFUs of a 1:1 mixture of the mutant and wild-type *P*. *mirabilis*, and 5x10^4^ CFUs of wild-type *P*. *stuartii*. In all cases, a 4 mm segment of catheter tubing was retained in the bladder for the duration of the study. Urine was collected and mice were sacrificed 4 days post-inoculation, and the bladder, kidneys, and spleen were homogenized and plated onto LB agar with and without kanamycin to determine bacterial burden of *P*. *stuartii*, wild-type *P*. *mirabilis*, and the mutant (A-C). Each data point represents the Log_10_ CFUs recovered from an individual mouse, and gray bars represent the median. A competitive index was calculated for each *P*. *mirabilis* mutant on a per-mouse basis using the ratio of mutant to wild-type in each organ divided by the ratio of mutant to wild-type from the inoculum to determine if the presence of *P*. *stuartii* resulted in the *P*. *mirabilis* mutant being significantly outcompeted by its parental wild-type strain (D-F). (A and D) the PMI1518 mutant vs wild-type *P*. *mirabilis* during coinfection with *P*. *stuartii*, (B and E) *lon*, and (C and F) *ilvD*. Solid lines represent the median. Dashed lines indicate a competitive index of 1, or a 1:1 ratio of mutant to wild-type. *P<0.05, **P<0.01, and ***P<0.001 by the Wilcoxon signed rank test.

To verify the coinfection Tn-Seq results, CBA/J mice were transurethrally inoculated with 1x10^5^ CFU of the following mixture: 5x10^4^ CFUs of a 1:1 mixture of the mutant and wild-type *P*. *mirabilis*, and 5x10^4^ CFUs of wild-type *P*. *stuartii*. CFUs of each species recovered from the urine, bladder, kidneys, and spleen are shown in Fig 10A–10C, and a competitive index was again calculated for each organ in which the mutant and wild-type were recovered above the limit of detection (Fig 11D–11F). As predicted from our Tn-Seq results, a mutant in the high-affinity nickel efflux protein encoded by PMI1518 was significantly outcompeted by wild-type *P*. *mirabilis* in the urine, bladder, kidneys, and spleen during coinfection with *P*. *stuartii*, indicating that nickel export is even more important to *P*. *mirabilis* during coinfection with *P*. *stuartii* than during single-species CAUTI (Fig 11D compared to Fig 8M). Also consistent with the Tn-Seq results, coinfection with *P*. *stuartii* appeared to alleviate the requirement for *lon*, as the mutant was no longer outcompeted by wild-type *P*. *mirabilis* in any organ during coinfection, and in some cases the *lon* mutant outcompeted wild-type by over 100-fold (Fig 11E). While *ilvD* was not important for *P*. *mirabilis* fitness during single-species infection, the *ilvD* mutant was significantly outcompeted by wild-type *P*. *mirabilis* in all organs during coinfection with *P*. *stuartii*, confirming that the presence of *P*. *stuartii* alters the metabolic requirements of *P*. *mirabilis*, resulting in a greater need for BCAA synthesis (Fig 11F). To verify that the fitness defect of this mutant was *in vivo* specific, co-challenge experiments were repeated *in vitro* in pooled urine from healthy human donors (S4 Fig). The *ilvD* mutant did not exhibit a significant competitive defect during growth with wild-type *P*. *mirabilis*, or in co-challenge during co-culture with wild-type *P*. *stuartii*. Thus, the requirement for *ilvD* during coinfection is specifically related to the host environment of the catheterized urinary tract and cannot be replicated in a test tube.

The Kyoto Encyclopedia of Genes and Genomes (KEGG) predicts that eight genes are required for synthesis of all three BCAAs in *P*. *mirabilis*, and five of these genes were candidate fitness factors during coinfection but only two appeared to contribute to fitness during single-species CAUTI (S9 Table). All four genes required for synthesis of leucine from 2-acetolactate (*leuABCD*) were fitness factors for coinfection but not for single-species infection, indicating a greater need for leucine biosynthesis during coinfection. Synthesis of isoleucine from threonine also appears to be important as all genes in this pathway were candidate fitness factors for coinfection. The BCAA transport system II carrier protein (*brnQ*) and two putative BCAA transporters encoded by PMI1599 and PMI1699 were also significant fitness factors for polymicrobial CAUTI but not single-species CAUTI, underscoring the importance of BCAAs to *P*. *mirabilis* fitness during coinfection. Taken together, these results indicate that *P*. *stuartii* may siphon away BCAAs within the urinary tract during coinfection, forcing *P*. *mirabilis* to rely more heavily on BCAA synthesis.

Unlike *P*. *mirabilis* HI4320, the genome of *P*. *stuartii* BE2467 encodes a high-affinity BCAA transporter (*livFGHM*) [21]. The Liv transporter utilizes two periplasmic binding proteins: LivJ for transport of all three BCAAs, and LivK for specific transport of leucine. As the *P*. *mirabilis* Tn-Seq data suggested a critical role for leucine biosynthesis in particular during coinfection, a *P*. *stuartii livK* mutant was generated to uncover the biological basis for the BCAA biosynthesis Tn-Seq results. Disruption of *livK* in *P*. *stuartii* did not impact growth in minimal medium compared to wild-type *P*. *stuartii*, although supplementation with excess leucine resulted in a slight perturbation of growth for wild-type *P*. *stuartii* that was not detected for the *livK* mutant (S5A Fig). We next wanted to verify that LivK was required for optimal growth of *P*. *stuartii* in minimal medium containing BCAAs when BCAA biosynthesis is inhibited. Copper sulfate has been shown to poison BCAA biosynthesis in *E*. *coli* [36]; we therefore supplemented minimal medium with 50 μM copper sulfate to inhibit *P*. *stuartii* BCAA biosynthesis and force *P*. *stuartii* to rely on uptake of exogenously-supplied BCAAs. In the absence of exogenous BCAAs, growth of the *livK* mutant and wild-type *P*. *stuartii* was perturbed to a similar extent by copper sulfate (S5B Fig). Growth of wild-type *P*. *stuartii* was partially rescued by 0.5 mM leucine and further increased by a 0.5 mM mixture of all three BCAAs, while growth of the *livK* mutant was not affected by leucine and only rescued when supplemented with all three BCAAs. Thus, *livK* is not required for *P*. *stuartii* growth in minimal medium, but contributes to leucine import.

**Fig 12:**
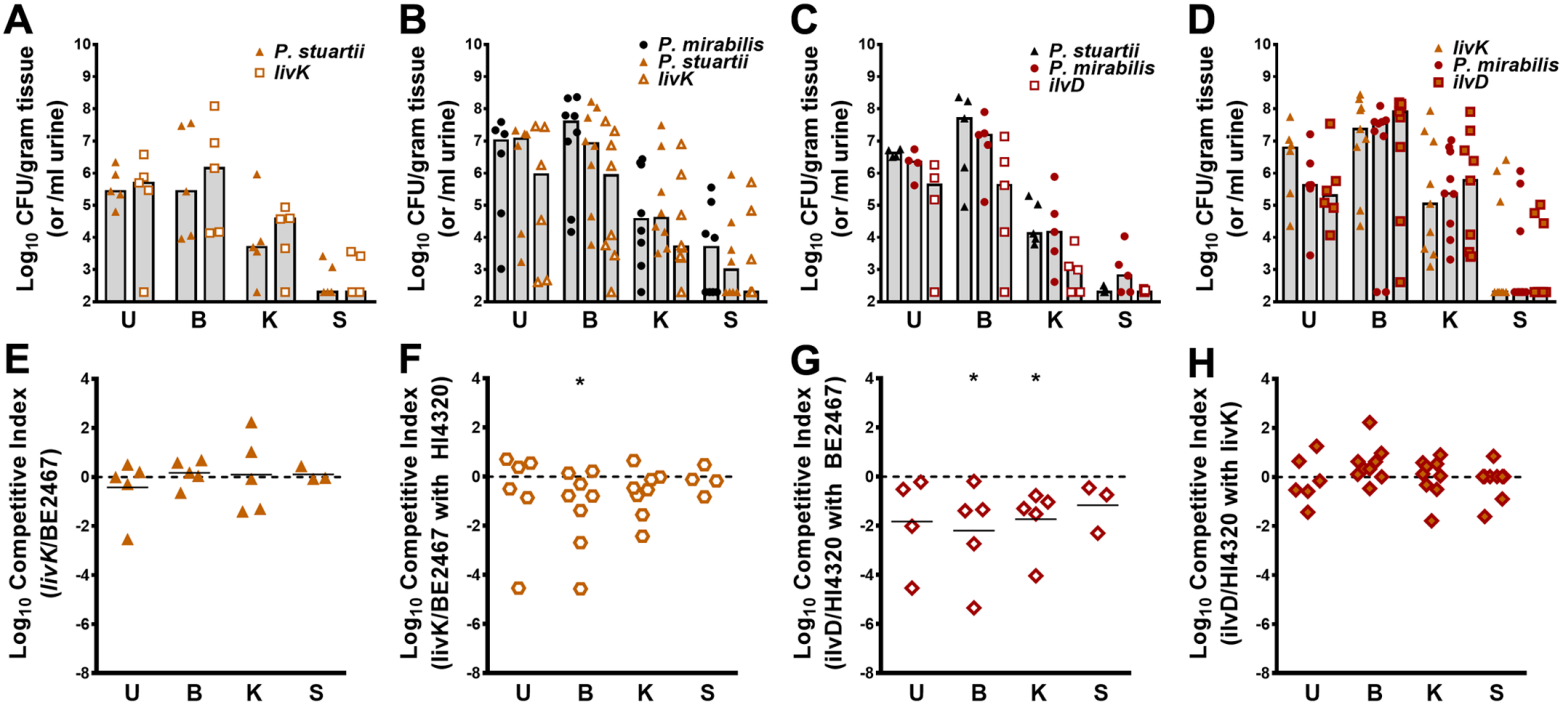
BCAA import and biosynthesis contribute to *P*. *stuartii* and *P*. *mirabilis* fitness during coinfection. CBA/J mice were transurethrally inoculated with 1x10^5^ CFU and a 4 mm segment of catheter tubing was retained in the bladder for the duration of the study. Urine was collected and mice were sacrificed 4 days post-inoculation, and the bladder, kidneys, and spleen were homogenized and plated onto LB agar with and without kanamycin to determine bacterial burden of *P*. *stuartii*, *P*. *mirabilis*, and their respective mutants (A-D), competitive indices were calculated for each infection (E-H). Mice were inoculated with the following mixtures: (A and E) 1:1 mixture of the *P*. *stuartii livK* mutant and wild-type *P*. *stuartii*, (B and F) 5x10^4^ CFU of a 1:1 mixture of the *P*. *stuartii livK* mutant and wild-type *P*. *stuartii* and 5x10^4^ CFU of wild-type *P*. *mirabilis*, (C and G) 5x10^4^ CFUs of a 1:1 mixture of the *P*. *mirabilis ilvD* mutant and wild-type *P*. *mirabilis* and 5x10^4^ CFUs of wild-type *P*. *stuartii*, and (D and H) 5x10^4^ CFUs of a 1:1 mixture of the *P*. *mirabilis ilvD* mutant and wild-type *P*. *mirabilis* and 5x10^4^ CFUs of the *P*. *stuartii livK* mutant. A competitive index was calculated for the *P*. *stuartii livK* mutant during single-species co-challenge (E) and co-challenge during coinfection with wild-type *P*. *mirabilis* (F), and a competitive index was calculated for the *P*. *mirabilis ilvD* mutant during coinfection with wild-type *P*. *stuartii* (G) or with the *P*. *stuartii livK* mutant (H). Error bars represent the median. Dashed lines indicate a competitive index of 1, or a 1:1 ratio of mutant to wild-type. *P<0.05 by the Wilcoxon signed rank test.

To determine if *livK* contributes to *P*. *stuartii* fitness during single-species or polymicrobial CAUTI, CBA/J mice were transurethrally inoculated with 1x10^5^ CFU of one of the following mixtures: 5x10^4^ CFUs of the *P*. *stuartii livK* mutant and 5x10^4^ CFUs of wild-type *P*. *stuartii* for a single-species co-challenge (Fig 12A and 12E), or 5x10^4^ CFUs of a 1:1 mixture of the *P*. *stuartii livK* mutant and wild-type, and 5x10^4^ CFUs of wild-type *P*. *mirabilis* for co-challenge during coinfection (Fig 12B and 12F). Interestingly, the *P*. *stuartii livK* mutant followed a similar trend as the *P*. *mirabilis ilvD* mutant, indicating that high-affinity leucine import only contributes to *P*. *stuartii* fitness during coinfection with *P*. *mirabilis*, but not during single-species infection. Finally, to determine if the requirement for BCAA synthesis by *P*. *mirabilis* during coinfection is primarily due to high-affinity leucine import by *P*. *stuartii*, the *P*. *mirabilis ilvD* co-challenge during coinfection studies were repeated using wild-type *P*. *stuartii* or the *livK* mutant. As previously observed in Fig 11F, the *P*. *mirabilis ilvD* mutant was outcompeted by wild-type *P*. *mirabilis* during coinfection with wild-type *P*. *stuartii* BE2467, again demonstrating the need for BCAA biosynthesis during coinfection (Fig 12C and 12G). However, the *P*. *mirabilis ilvD* mutant was no longer outcompeted by wild-type *P*. *mirabilis* during coinfection with the *P*. *stuartii livK* mutant (Fig 12D and 12H), indicating that high-affinity leucine import by *P*. *stuartii* induces the need for BCAA synthesis by *P*. *mirabilis* during coinfection. We therefore conclude that coinfection measurably alters *P*. *mirabilis* fitness requirements for CAUTI, and that our infection model can be used to examine the interplay between fitness requirements for both species during coinfection.

## Discussion

*Proteus mirabilis* HI4320 is a model organism that has been used for decades to explore virulence determinants of this unusual bacterial species. With the availability of the complete genome sequence in 2008 [26], prior signature-tagged mutagenesis studies [17–19], and *in vivo* transcriptome assessment [31], much has been learned concerning how this organism differentiates into swarm cells, how it survives in the environment and during infection, the metabolic processes that allow for adaptation to the bladder and kidney environments, and virulence factors that contribute to ascending UTI (see [2, 16] for review). Despite this wealth of information, numerous questions remained regarding global *P*. *mirabilis* fitness and virulence factors, particularly concerning the relevance of known fitness factors to catheter-associated infection.

The present study details the first use of Tn-Seq for identification of fitness factors in a murine model of catheter-associated urinary tract infection (CAUTI), as well as the first use of Tn-Seq during polymicrobial infection. Strengths of this study include: 1) the use of multiple transposon library pools and four “replicate” mice per infection pool, 2) stringent cutoffs for analysis of insertion-site reads, and 3) generation and testing of mutants in each of the bacterial species under investigation for mechanistic insight into altered fitness requirements during coinfection. It is important to note that our approach is restricted by a few caveats common to all Tn-Seq studies, including an inability to assess the fitness contribution of genes for which insertions were not present in the input pools and difficulty assessing the fitness contribution of secreted factors due to complementation by other mutants within the input pools. Another limitation of the study comes from the use of two urease-positive organisms for our CAUTI coinfection model: the catheter segments were generally embedded in the bladder tissue, so we were unable to remove them for a separate assessment of fitness factors for catheter colonization versus bladder colonization. Thus, the fitness factors for bladder colonization likely represent a combination of factors for colonization of the catheter segment as well as the bladder epithelium.

Transposon insertions may also have polar effects on genes that are within an operon, a consideration that needs to be explored for follow-up studies on fitness factors of interest. For the eight mutants used for validation of the primary screen, four are within operons. The transcription unit for *hslU* includes *hslV*, the other subunit of the ATP-dependent protease that was similarly found to be a fitness factor. The transcription unit for PMI1518 includes PMI1519, encoding a hypothetical substrate binding protein. PMI1519 is upstream of PMI1518 in the transcription unit, and this gene contained transposon insertions but was not identified as a fitness factor, indicating the central importance of the efflux protein encoded by PMI1518. For *ilvD*, the transcription unit is organized as *ilvGMEDA*, and there were differences in the fitness defects for each of these genes as show in S9 Table. ArnA also resides in the middle of a transcription unit, but the defects for this operon are not likely due to polar effects as the first four genes were fitness factors for single-species CAUTI but not during coinfection, and the remaining three genes in the operon were identified as essential for growth in rich medium.

Despite these limitations, our approach uncovered numerous previously unrecognized *P*. *mirabilis* fitness determinants and explored the impact of polymicrobial infection on fitness requirements. Indeed, fitness defects were verified for 7/8 (88%) candidate fitness factors tested by direct co-challenge with wild-type *P*. *mirabilis* and 3/3 mutants tested during coinfection. Results of interest from the essential gene analysis, single-species CAUTI, and polymicrobial CAUTI are detailed below.

### *P*. *mirabilis* genes essential for growth in rich medium

The majority of the large regions of the chromosome that lacked transposon insertions and were visible as gaps in Fig 1 did indeed correspond to genes identified as essential. For instance, the largest region that lacked transposon insertions was 12,423 bp encompassing 18 genes involved in cell division and cell wall biogenesis. The next largest regions that lacked transposon insertions were a 7,543 bp region encompassing genes necessary for translation, and a 6,650 bp region encompassing an intergenic region and *murI*, a glutamate racemase that is essential for cell wall biosynthesis. However, this was not always the case for gaps in transposon insertions on plasmid pHI4320. The largest region lacking insertions on the plasmid was 1,252 bp encompassing an intergenic region and a portion of PMIP30, which encodes a putative colicin. This gene was not identified as essential as there were 81 insertion sites after the gap which were well-represented in the input pools. Another gap on the plasmid of 826 bp corresponds to a portion of PMIP42, a putative RelB antitoxin. Similar to PMIP30, PMIP42 contained two insertion sites after the gap that were highly represented in the input pools, which likely caused it to fall below the threshold for being considered essential. Thus, the model used to identify putative essential genes provided very conservative estimates, and is likely an underrepresentation of the genes required for optimal growth of *P*. *mirabilis* in LB. It is therefore notable that the other ~500 bp gaps in transposon insertions on the plasmid were within a conjugal transfer protein (PMIP09), a colicin immunity protein (PMIP31), a plasmid stability/partitioning protein (PMIP34), a type IA DNA topoisomerase (PMIP25), and HN-S family protein (PMIP26). These findings are in agreement with the replication initiation protein (PMIP01) being essential, and underscore the importance of plasmid maintenance and replication to *P*. *mirabilis* growth.

In addition to the numerous genes previously identified as essential for growth in other bacterial species, the list of *P*. *mirabilis* estimated essential genes contained a few unusual items. For instance, all but one of the genes in the non-oxidative pentose phosphate pathway were identified as putative essential genes (*rpiA*, *rpe*, *tktA*, and *talB*), indicating a central role for this pathway during growth of *P*. *mirabilis* in rich medium. Phosphoglycerate kinase (*pgk*), which is involved in glycolysis, gluconeogenesis, and glycerol degradation, was also identified as a possible essential gene. Factors involved in inorganic ion transport and metabolism were a surprising find, as these genes are not commonly identified as essential. These included genes involved in potassium uptake (*trkA* and *trkH*), intracellular sulfur oxidation (PMI2797 and PMI2798), tellurite resistance (*terC*), a sulfite reductase (PMI0794), and magnesium and cobalt efflux (*corC*). TrkA and TrkH comprise a high-rate low-affinity potassium-translocating system that requires ATP via the Sap system [37], and all members of the Sap dipeptide transport system transcriptional unit (*sapBCDF*) were also identified as putative essential genes.

Six fimbrial genes were also identified as potential essentials, including three homologs of the *mrpJ* fimbrial operon regulator [38]. Little is known concerning the identified fimbrial genes (*pmpB*, *fim3J*, *fim5G*, *fim7D*, *fim8J*, *fim10D*, and *fim10J*), or why disruption of these genes would impact growth in rich medium. Three of these fimbrial genes encode homologs of *mrpJ*, a transcriptional regulator that represses motility but also influences expression of other adhesins, virulence factors, and metabolic pathways [39]. Homologs of *mrpJ* are also capable of regulating motility and adherence [38], so it is possible that these *mrpJ* homologs similarly play a role in a broad regulatory network that could contribute to growth, or that tight regulation of the expression of these operons is important during growth in rich medium. Two homologs of the type VI secretion system (T6SS) secreted protein Hcp (PMI0750 and PMI1117) were also identified as putative essential genes, as well as 17 transposases and 1 phage repressor protein. In each of these cases, the identified genes contained >15 TA sites for transposon insertion, but <3 total insertions were recovered from the five combined input pools. While these genes may be important for growth in rich medium, it is also possible that the mariner transposon wasn’t able to efficiently target these chromosomal locations. Thus, the importance of the unique genes estimated to be essential for *P*. *mirabilis* should be interpreted with caution.

### Validation of known *P*. *mirabilis* fitness factors for single-species infection

#### Candidate fitness factors from signature-tagged mutagenesis studies

Several of the candidate fitness factors for both bladder and kidney colonization were previously identified in STM studies of ascending UTI [17–19], as mentioned in the Results section. Included in this list is the bifunctional polymyxin resistance protein (*arnA*). ArnA and ArnB provide resistance to polymyxin B and other cationic antimicrobial peptides due to their role in lipid A modification with 4-amino-4-deoxyl-l-arabinose (l-Ara4N) [40], so it is not surprising that all genes responsible for conversion of UDP-glucuronic acid (UDP-GlcA) to undecaprenyl phosphate-α- l-Ara4N (*arnABC* and PMI1046) were candidate fitness factors during single-species CAUTI. The contribution of ArnA to CAUTI was validated in our experimental model as the *arnA* mutant was outcompeted by wild-type *P*. *mirabilis* in the bladder, kidneys, and spleen during direct co-challenge, underscoring the importance of resistance to antimicrobial peptides for fitness within the urinary tract.

#### Fimbriae

Fimbriae represent major fitness factors for *P*. *mirabilis* colonization during ascending UTI [2, 16, 41]. The uroepithelial cell adhesin (UCA, also known as NAF for nonagglutinating fimbriae) contributes to ascending UTI [42], and both *ucaA* and *ucaB* were identified as fitness factors for CAUTI. *P*. *mirabilis* fimbriae (PMF) have been reported to contribute to colonization during ascending UTI [43], and this holds true for CAUTI as *pmfA* was identified a fitness factor. Although the *pmp* fimbrial operon has not been well-characterized, the putative fimbrial protein encoded by PMI2217 (*pmpG*) was a fitness factor for *P*. *mirabilis* during single-species CAUTI. Other fimbriae that contribute to colonization during ascending UTI are the mannose-resistant *Proteus-*like (MRP) fimbriae [41]; however, the only *mrp* genes identified as candidate fitness factors for CAUTI were from a duplication of the *mrp* operon annotated as *mrp`*. As the *mrp`*operon has not previously been shown to contribute to fitness, these data clearly indicate that the presence of the catheter influences the adhesion requirements of *P*. *mirabilis*.

### Differences in *P*. *mirabilis* fitness requirements for single-species CAUTI vs ascending UTI

#### Metabolism

One of the most striking differences in fitness requirements for CAUTI compared to UTI relates to the metabolic pathways favored by *P*. *mirabilis* in each infection model. Glycolysis, the oxidative pentose phosphate pathway, and the Entner-Doudorof pathway were previously determined to contribute to *P*. *mirabilis* fitness in ascending UTI, as mutants in these pathways were outcompeted by the wild-type strain [44]. However, none of these pathways were identified as important for colonization during single-species CAUTI by Tn-Seq, with the exception of pyruvate kinase (*pykF*), which catalyzes the transfer of a phosphate group from phosphoenolpyruvate to yield ATP and pyruvate. The cytochrome *bo*3 quinol oxidase (*cyoABCD*) was also identified as a fitness factor for both bladder and kidney colonization. Considering that cytochrome quinol *bo* oxidases generally reduce O_2_ to H_2_O, this finding indicates that *P*. *mirabilis* is likely utilizing aerobic respiration during CAUTI.

In the present Tn-Seq study, it appears that *P*. *mirabilis* could derive energy from breaking down peptides and amino acids present in the inflammatory bladder environment during CAUTI. This hypothesis is supported by the substantial number of fitness factors pertaining to amino acid transport and metabolism that were identified as important for both bladder and kidney colonization. For instance, d-serine exists at high concentrations in urine relative to other amino acids [45, 46], and d-serine dehydratase (*dsdA*, which generates pyruvate and ammonia from d-serine) was a *P*. *mirabilis* fitness factor for both bladder and kidney colonization during CAUTI.

Prior work in the ascending UTI model indicated a central role for nitrogen assimilation via glutamate dehydrogenase [31], and it was speculated that *gdhA* was required due to the excess of nitrogen that occurs from the action of urease [44]. However, this does not appear to be the case during CAUTI as *gdhA* was not identified as a candidate fitness gene in the Tn-Seq screen. Glutamine synthetase (*glnA*), on the other hand, was identified as a fitness factor for CAUTI, and the importance of this gene to *P*. *mirabilis* fitness was confirmed by direct co-challenge with wild-type *P*. *mirabilis*. Thus, there appears to be a differential requirement for metabolic pathways as well as nitrogen assimilation pathways during UTI and CAUTI.

#### Flagella and swarm cell differentiation

Two genes in the flagellar regulon (*fliF* and *flgE*) were previously identified as fitness factors for ascending UTI by STM [19], but there has been only limited evidence to suggest a direct role for flagella in *P*. *mirabilis* pathogenesis within the urinary tract [19, 47–49]. The flagellar components encoded by *fliF*, *fliI*, and *flgC* were identified as fitness factors for CAUTI, along with the master regulator *flhDC* and numerous swarming-related genes. These include the Lon protease (*lon*, which degrades FlhD [50]), CsrA (a positive regulator of *flhDC* [51]), as well as UmoA and UmoB (which regulate *flhDC* [52, 53]). Taken together, these data suggest a role for flagella-mediated motility, and possibly even swarm-cell differentiation, in the CAUTI model. Zinc uptake is also important for swarming motility [54], so the identification of the *ppaA* and *znuABC* zinc transporters as CAUTI fitness factors is in agreement with this hypothesis.

#### Toxins and metal acquisition

*Proteus* toxic agglutinin (Pta) and hemolysin (*hpmA*) contribute to fitness in the ascending UTI model [55], yet neither was identified as a significant fitness factor during single-species CAUTI. *P*. *mirabilis* may not require as many toxins in the CAUTI model due to the greater level of inflammation and tissue damage induced by maintenance of the catheter segment in this infection model [21]. Regarding metal acquisition, proteobactin and the yersiniabactin-related siderophore (encoded by the *nrp* operon) were previously shown to be important for ascending UTI [56], but neither were fitness factors during CAUTI. Similarly, the heme receptor *hmuR2* contributes to colonization during ascending UTI [57] but was not identified as a fitness factor for single-species CAUTI.

### Novel fitness factors for *P*. *mirabilis* single-species infection

#### Energy-dependent proteases

Three of the five energy-dependent proteases encoded by *P*. *mirabilis* were estimated to be essential genes (HflB, ClpAP, and ClpXP). The remaining proteases (ClpYQ and Lon) have not previously been explored for a role during UTI, but both were identified as candidate fitness factors for single-species CAUTI. The Lon protease degrades naturally unstable proteins as well as misfolded proteins that would otherwise aggregate [30], and both Lon and ClpYQ can act on SulA (an SOS regulon constituent that inhibits cell division) [30]. Considering that both the *lon* mutant and the *hslU* (ClpY) mutant exhibited fitness defects during direct co-challenge with wild-type *P*. *mirabilis*, the importance of these energy-dependent proteases and their targets to *P*. *mirabilis* fitness and pathogenicity warrants further exploration.

#### Toxin/Antitoxin systems

Strikingly, a putative HicA toxin (PMI0818) was the number one single-species infection fitness factor in Tables 1 and 2, exhibiting a 28-fold defect in the bladder and a 19-fold defect in the kidneys. The HicB antitoxin (PMI0817) was the number two fitness factor for bladder colonization (23-fold defect) and was also a fitness factor for kidney colonization (~5-fold). The HicAB system is present on phages and plasmids and is capable of arresting growth in plasmid- or phage-free cells, and these systems have been found to be integrated into the chromosome of numerous representatives of the major clades of bacteria and archaea [58], as is the case for *P*. *mirabilis* HI4320.

When bacterial cells are growing normally, the toxins are neutralized by the binding of their co-transcribed antitoxins. For chromosomal toxin/antitoxin systems, degradation of the antitoxin is often induced by nutritional or environmental stress, and these systems are hypothesized to be involved in programmed bacterial cell death [59, 60]. In *E*. *coli* K-12, transcription of *hicAB* is induced by amino acid or carbon starvation [61]. HicAB has also been implicated in persister cell formation during antibiotic exposure [62]. Most importantly for the present study, the antitoxin HicB appears to be subject to degradation by the Lon protease in *E*. *coli* K12 under starvation conditions, resulting in de-repression of the HicA toxin [61]. Thus, it is notable that the energy-dependent proteases encoded by *lon* and *hslU* follow the same pattern as *hicAB* in the present Tn-Seq study. Furthermore, it has been demonstrated that toxins from one toxin/antitoxin pair can activate transcription of other toxin/antitoxin operons [63], and antitoxins may also repress a variety of other promoters in addition to the toxin/antitoxin system [64]. The antitoxin of the ParDE system (PMI0550) was another top fitness factor for both bladder and kidney colonization, supporting the potential role for crosstalk between toxin/antitoxin systems in *P*. *mirabilis*. Further research will be necessary to explore the underlying regulatory network of energy-dependent proteases and the multiple toxin/antitoxin systems encoded by *P*. *mirabilis*.

#### Stress responses

Several of the candidate fitness genes for both bladder and kidney colonization indicated a role for adaptation to stress during CAUTI, possibly due to the pro-inflammatory environment induced in this model. These include cold-shock proteins (*cspC*, *cspE*, and *cspC*), heat-shock proteins (*clpB*), phage-shock proteins (*pspA*), universal stress proteins (*uspA* and *uspG2*), the carbon storage regulator *csrA*, chaperones (*hscAB*), and DNA-binding protein HU (*hupAB*). Several of the fitness factors indicate that *P*. *mirabilis* may need to adapt to oxidative stress during CAUTI, including superoxide dismutase (*sodA*), the redox-sensitive transcriptional activator *soxR*, iron regulatory proteins *iscA*, *iscU*, and *iscR*, ferric uptake regulator *fur*, and fumarate and nitrate reduction regulator *fnr*. Interestingly, *oxyR* was not a candidate fitness gene for single-species CAUTI, and catalase (*katA*) was only identified as a candidate fitness factor for bladder colonization. Thus, it may be more critical for *P*. *mirabilis* to adapt to superoxide and nitric oxide stress than hydrogen peroxide. Further research will be necessary to characterize the *P*. *mirabilis* stress response during CAUTI.

#### Putrescine

Arginine decarboxylase (*speA*) catalyzes the first step in the primary pathway for putrescine biosynthesis from l-arginine in *P*. *mirabilis* [32]. This reaction contributes to both the production of putrescine, known to play a critical role in *P*. *mirabilis* swarming [32, 65, 66], as well as the consumption of intracellular protons, thereby contributing to maintenance of membrane potential, proton motive, and survival in mildly acidic conditions such as would be encountered within the urinary tract [67]. In the CAUTI model, genes involved in putrescine biosynthesis from l-arginine (*speA*, *speB*) and putrescine import (*plaP* and *potABCD*) were fitness factors for single-species infection. In contrast, none of the genes involved l-arginine biosynthesis or import were identified as fitness factors for CAUTI, but the repressor of arginine biosynthesis (*argR*) was verified to contribute to fitness during direct co-challenge with wild-type *P*. *mirabilis*. It is therefore likely that the importance of *speA* in this infection model is due to its role in putrescine biosynthesis, in contrast to its role in maintenance of proton motive force during ascending UTI [67].

### Conserved fitness requirements for *P*. *mirabilis* single-species and polymicrobial CAUTI

#### Core fitness requirements

Fifteen fitness factors were identified for all conditions tested (bladder and kidney colonization for both single-species and polymicrobial infection), and therefore hypothesized to represent “core” fitness factors for *P*. *mirabilis*. Two of these genes (PMIP02 and PMIP15) are encoded on the 36 Kb extra-chromosomal plasmid of *P*. *mirabilis* HI4320 (pHI4320), indicating a critical role for plasmid-encoded factors in *P*. *mirabilis* fitness. Another gene, PMI0621 (*dusC*) encodes a flavin-dependent tRNA-dihydrouridine synthase (Dus). This enzyme catalyzes the synthesis of 5,6-dihydrouridine, a highly conserved modified base and one of the most common post-transcriptional modifications of tRNA. *E*. *coli* K-12 encodes three Dus homologs, which act in a site-specific manner on the tRNA D-loop [68]. *P*. *mirabilis* HI4320 similarly encodes three Dus homologs (*dusA*, *dusB*, and *dusC*) [26]. DusB was previously identified as fitness factor for *P*. *mirabilis* by STM in the ascending UTI model [18], and *dusC* represents a candidate fitness factor for bladder and kidney colonization in all 40 mice from the present study (single-species and coinfection). Thus, 5,6-dihydrouridine appears to be critical for fitness during both UTI and CAUTI, possibly due to its role in tRNA stability.

#### Concordance between *P*. *mirabilis* fitness factors for single-species and polymicrobial CAUTI

217 out of the 629 genes identified as fitness factors for single-species CAUTI (34.5%) were also fitness factors for polymicrobial CAUTI, indicating substantial overlap in fitness requirements for *P*. *mirabilis* during these two infection types. Among this category of fitness factors are fimbrial genes (*mrpA`*and *mrpF`*from the *mrp`* operon, and *pmpG*), components of the flagellar cascade and regulation of motility (*flhC*, *fliF*, *fliI*, *cheR*), and components of the urease operon (*ureG* for single-species infection and *ureRDCF* for polymicrobial infection). Numerous factors pertaining to inorganic ion transport and metabolism were also conserved between infection types: the *hmuS* hemin transporter protein, the *ireA* siderophore receptor, a copper homeostasis protein (*cutC*), a molybdenum ABC transporter permease protein (*modB*), and the high-affinity nickel transporter encoded by PMI1518. The contribution of PMI1518 to both single-species and polymicrobial CAUTI was verified during direct co-challenge with the wild-type *P*. *mirabilis*, providing the first evidence that nickel homeostasis directly contributes to *P*. *mirabilis* fitness during CAUTI.

### *P*. *mirabilis* fitness requirements unique to polymicrobial CAUTI

#### Defense mechanisms

Genes that could provide *P*. *mirabilis* with a competitive advantage against another species, such as toxins and secretion systems, were not significant fitness factors for single-species CAUTI but were important during polymicrobial infection with *P*. *stuartii*. These include the *Proteus* toxic agglutinin (Pta), hemolysin (*hpmA*), and numerous multidrug resistance proteins and efflux pumps pertaining to the “defense mechanisms” COG. Two members of the type III secretion system (T3SS) in *P*. *mirabilis* (*spa24*, and *mxiD*) were significant fitness factors for coinfection and not during single-species infection, which is consistent with a prior study indicating that T3SS does not contribute to *P*. *mirabilis* virulence during single-species UTI [69].

Most striking, however, was the observation that all of the known *P*. *mirabilis* HI4320 type VI secretion system (T6SS) operons were overrepresented as candidate fitness factors specifically during coinfection; the identification of self operon (*idsABCDEF*) [70, 71], the primary effector operon (*pefABCDEF*) [72], and four additional operons (PMI0207-PMI0212, PMI0749-PMI0734, PMI1117-PMI1121, and PMI1332-PMI1324)[26, 72]. This finding clearly indicates a role for T6S in mediating competitive and cooperative interactions during polymicrobial infection, either between *P*. *mirabilis* and *P*. *stuartii* or between *P*. *mirabilis* and the host in the altered urinary tract environment that occurs during coinfection. This is particularly notable as the genome of *P*. *stuartii* BE2467 similarly encodes a putative T6SS effector operon and several Vgr homologs [21]. T6S-mediated interactions between these organisms and other uropathogens warrants further exploration.

#### Signal transduction

Coinfection with *P*. *stuartii* resulted in the requirement of numerous two-component systems and transcriptional regulators for *P*. *mirabilis* fitness, many of which have not been characterized or are only annotated as putative regulatory proteins. Notable two-component regulators include the regulator of capsular synthesis (*rcsBC*) and the nitrate/nitrite two-component system (*narXL*), a phosphoglycerate transport regulator and sensor kinase (*pgtAB*), members of the phosphate regulon (*phoB*, *phoR*, and *phoQ*), the potassium transport sensor protein (*kdpD*), and a sensor kinase involved in regulation of swarming motility (PMI1697). Investigation of the exact contribution of these systems to *P*. *mirabilis* fitness during polymicrobial CAUTI may uncover polymicrobial interactions that modulate *P*. *mirabilis* virulence.

#### Fimbriae

The genome of *P*. *mirabilis* HI4320 encodes 17 distinct fimbrial operons, only five of which have been characterized in any *in vivo* model [16]. While some of these fimbriae were fitness factors for single-species CAUTI, such as the *mrp`*operon, several fimbrial genes were only identified as fitness factors during polymicrobial infection, including the main *mrp* operon, the ambient-temperature fimbriae, *fim3*, *fim5*, *fim12*, *fim14*, *fim15*, *fim16*, *fim17*, and putative fimbrial proteins encoded by PMI1812, PMI1920, and PMI3023. Thus, coinfection with *P*. *stuartii* appears to alter adhesion requirements for *P*. *mirabilis*, or may expose additional binding substrates that do not provide an advantage during single-species CAUTI.

#### Inorganic ion transport and metabolism

The coinfection Tn-Seq results indicate that *P*. *stuartii* influences *P*. *mirabilis* metal homeostasis and the metabolism and transport of numerous ions. For instance, several genes involved in iron acquisition and storage were overrepresented as fitness factors during coinfection, such as the genes encoding proteobactin (*pbtABCDEFGH*, *pbtI*), the yersiniabactin-related siderophore (*nrpXYRSUTABG*), the Sit iron ABC transporter (*sitABCD*), hemin receptors (*hmuR1* and *hmuR2*) and permease (*hmuU*), and ferritin (*ftnA*), indicating a more pressing need for iron scavenging during competition with another bacterium. Additional coinfection-specific fitness factors from this category include seven TonB-dependent receptors, two components of a molybdate ABC transporter, and putative sulfate and thiosulfate transporters. However, it has yet to be determined if the altered requirement for these genes by *P*. *mirabilis* is in direct response to *P*. *stuartii*, or to differences in the host urinary tract environment during coinfection compared to single-species infection.

#### Energy production and respiration

Due to the requirement of the aerobic cytochrome *bo*3 during single-species *P*. *mirabilis* CAUTI, it is noteworthy that the respiratory nitrate reductase system (*narGHJI*), the *narXL* two-component sensor kinase, the *narP* response regulator, the *narK* nitrite extrusion protein, the *nirB* nitrite reductase, and the flavohemoprotein *hmp* were all identified as candidate *P*. *mirabilis* fitness factors during coinfection but not single-species infection. This may be a reflection of ammonia accumulation from enhanced urease activity during coinfection or the increased inflammation during coinfection [21], either of which could contribute to oxidative stress and drive a greater requirement for anaerobic nitrate and nitrite reduction. The Rnf redox-driven ion pump (*rnfABCDGE*), which may play a role in reducing SoxR [73], was also identified as important during coinfection but not single-species infection. Catalase (*katA*) was another candidate fitness factor during coinfection, further suggesting that coinfection places *P*. *mirabilis* under greater oxidative stress than single-species CAUTI.

#### Metabolic pathways

Consistent with prior studies of ascending UTI [44], the metabolic pathways favored by *P*. *mirabilis* were impacted by polymicrobial infection in the CAUTI model. The oxidative pentose phosphate pathway and the Entner-Doudorof pathway were not identified as being important for colonization during single-species CAUTI, but both pathways were fitness factors during coinfection with *P*. *stuartii*. For the oxidative pentose phosphate pathway, this includes glucose-6-phosphate 1-dehydrogenase (*zwf*), 6-phosphogluconolactonase (*pgi*), and 6-phophogluconate dehydrogenase (*gnd*). For the Entner-Doudorof pathway, this includes phophogluconase dehydratase (*edd*) and KHG/KDPG aldolase (*eda*).

#### Arginine and putrescine

All genes pertaining to the l-arginine biosynthetic pathway (*argA*, *argCBGH*, *argE*, and *argI*) and the arginine ABC importer (*artPIQM*) were fitness factors for polymicrobial CAUTI but not during single-species infection. This finding is in agreement with *argR*, a repressor of arginine biosynthesis, only having a significant fitness defect during single-species infection. Similarly, none of the genes involved in putrescine biosynthesis from l-arginine (*speA*, *speB*) or import (*plaP* and *potABCD*) were fitness factors during polymicrobial CAUTI, despite their role during single-species infection. Taken together, these data suggest that the presence of *P*. *stuartii* stimulates *P*. *mirabilis* to rely more heavily on arginine uptake and biosynthesis, while alleviating the requirement for polyamine uptake and biosynthesis.

#### Branched chain amino acid (BCAA) synthesis

All four *P*. *mirabilis* genes required for leucine biosynthesis from 2-acetolactate (*leuABCD*) were fitness factors during polymicrobial CAUTI but not single-species infection, and an *ilvD* mutant unable to make BCAAs was significantly outcompeted by wild-type *P*. *mirabilis* only during coinfection with *P*. *stuartii*. As *P*. *stuartii* encodes a high-affinity BCAA transporter, we hypothesized that this unique fitness requirement during coinfection could be due to *P*. *stuartii* siphoning away BCAAs within the urinary tract, forcing *P*. *mirabilis* to rely more heavily on BCAA synthesis. Indeed, high-affinity leucine import by *P*. *stuartii* only contributed to fitness during coinfection with *P*. *mirabilis*. Similarly, *P*. *mirabilis* only required BCAA synthesis for fitness during coinfection with wild-type *P*. *stuartii* and not the *P*. *stuartii livK* mutant. These findings are of particular interest as BCAAs are essential amino acids in humans, making BCAA biosynthesis a potential target for therapeutic intervention, particularly if biosynthesis is an important fitness requirement for multiple species during polymicrobial infection. Indeed, BCAA synthesis has been shown to be important for other species in other infection models, including *Klebsiella pneumoniae* [74].

### *P*. *mirabilis* fitness requirements complemented during polymicrobial CAUTI

As mentioned above, the genes involved in putrescine uptake and biosynthesis were only identified as fitness requirements for *P*. *mirabilis* single-species CAUTI and not polymicrobial CAUTI, indicating that the presence of *P*. *stuartii* alleviates the polyamine requirement of *P*. *mirabilis*. The same appears to be true of the high-affinity zinc transport system encoded by *znuABC*. Stress responses also appear to be more important during single-species CAUTI than coinfection, as the majority of the stress-related genes identified as *P*. *mirabilis* fitness factors for single-species CAUTI were not significant during coinfection. Similarly, glutamine synthetase (*glnA*) was important for single-species fitness but not coinfection, further underscoring the differences in metabolic pathways favored by *P*. *mirabilis* during these two infection types. Further research is needed to determine which of these shifts in fitness requirements are specifically due to *P*. *stuartii* and which are due to the altered host environment and response to coinfection compared to *P*. *mirabilis* single-species infection.

### Transposon insertions that produce a fitness defect during single-species CAUTI and a possible advantage during polymicrobial CAUTI

#### Energy-dependent cellular functions

Many of the 109 genes for which transposon insertion resulted in a fitness defect during single-species infection but an apparent advantage during coinfection pertain to important cellular functions that also require a substantial amount of energy to produce or to function. Thus, loss of these genes could allow for a competitive advantage. This category includes the cytochrome *bo*3 quinol oxidase genes (*cyoABCD*), the phosphate ABC transporter (*pstSCAB*), and the ATP-dependent proteases (ClpYQ and Lon). Energy-dependent protein degradation is very expensive to a bacterial cell, requiring anywhere from 20 to >500 ATP molecules [75]. Mutation of an energy-dependent protease may therefore provide a competitive growth advantage under the right conditions, particularly if another protease has overlapping substrates. Indeed, coinfection with *P*. *stuartii* clearly alleviated the fitness defect of the *lon* mutant, and while the mutant did not significantly outcompete wild-type *P*. *mirabilis*, there was a trend towards the *lon* mutant exhibiting a competitive advantage in several of the mice.

#### Toxin/Antitoxin systems

The HicAB toxin/antitoxin system was the number one fitness factor for bladder colonization during single-species CAUTI. However, transposon insertion in the HicA toxin (PMI0818) resulted in a 2-10-fold advantage during coinfection in our Tn-Seq screen, and the fitness requirement for the HicB antitoxin appeared to be complemented by coinfection with *P*. *stuartii*. This finding is in agreement with the role of the Lon protease during infection, and may indicate that HicB is a substrate of Lon in *P*. *mirabilis*. Further research is needed for verification and to determine if loss of this system provides an advantage during coinfection due to the toxin system itself, other regulatory targets of the toxin and antitoxin, or the energy requirement for the production and function of the system.

### Conclusion

The combination of genome-saturating transposon mutant libraries and Tn-Seq has allowed for the first global estimation of *P*. *mirabilis* essential genes, validation of numerous *P*. *mirabilis* virulence factors and fitness determinants from decades of studies using the ascending model of UTI, the discovery of novel fitness determinants specifically for CAUTI, and a stringent assessment of how polymicrobial infection influences fitness requirements. For instance, proteobactin, a yersiniabactin-related siderophore (*nrp*), and heme receptors were promising targets for perturbing *P*. *mirabilis* ascending UTI [56], but these factors do not appear to be important for fitness during single-species infection in the CAUTI model. BCAA biosynthesis is an intriguing target for reducing bacterial colonization, but our results indicate that this pathway is only important for *P*. *mirabilis* during polymicrobial infection and therefore would not be a suitable target for single-species infection by *P*. *mirabilis*. In contrast, our data indicate that polyamine uptake and biosynthesis may be promising targets for perturbing *P*. *mirabilis* single-species CAUTI, but these pathways were not important during coinfection, which may limit their potential as therapeutic targets given the high frequency of *P*. *mirabilis* polymicrobial colonization and infection.

Further research is needed concerning the numerous genes and pathways that provided a competitive advantage to *P*. *mirabilis* during both single-species and polymicrobial CAUTI, as the underlying mechanisms of these fitness requirements are the most likely to provide conserved targets for therapeutic intervention aimed at reducing *P*. *mirabilis* colonization or minimizing risk of progression to severe infection and urosepsis. We have clearly demonstrated that the CAUTI coinfection model can be used to examine the interplay between fitness requirements for both species during coinfection. It is therefore likely that investigation of the fitness requirements of other common CAUTI pathogens for single-species and polymicrobial CAUTI will further elucidate complex bacterial interactions that contribute to disease severity, and may even uncover conserved bacterial targets for therapeutic intervention.

## Materials and methods

### Ethics statement

#### Animal protocols

All animal protocols were approved by the Institutional Animal Care and Use Committee (IACUC) at the University of Michigan Medical School (PRO00005052), in accordance with the Office of Laboratory Animal Welfare (OLAW) and the United States Department of Agriculture (USDA), as well as guidelines specified by the Association for Assessment and Accreditation of Laboratory Animal Care, International (AAALAC, Intl.). Mice were anesthetized with a weight-appropriate dose (0.1 ml for a mouse weighing 20 gm) of ketamine/xylazine (80–120 mg/kg ketamine and 5–10 mg/kg xylazine) by IP injection. Mice were euthanized by inhalant anesthetic overdose followed by vital organ removal.

#### Clinical isolates and human urine

The wild-type bacterial isolates used in this study were collected in Baltimore, Maryland in a prior study [4, 76]. All isolates were collected with consent and were anonymized. Urine collection for use as a growth medium was performed as approved by the University of Michigan Institutional Review Board (HUM00004949) from anonymized healthy human subjects at the University of Michigan.

#### Bacterial strains and culture conditions

*Proteus mirabilis* HI4320 and *Providencia stuartii* BE2467 were isolated in a prior study from the urine of catheterized patients in chronic care facilities in Baltimore, Maryland [4, 76]. Bacteria were routinely cultured at 37°C with aeration in 5 ml LB broth (10 g/L tryptone, 5 g/L yeast extract, 0.5 g/L NaCl) or on LB solidified with 1.5% agar. Swarming motility was assessed using swarm agar (LB agar with 5 g/L NaCl). Swimming motility was assessed using Mot medium (10 g/L tryptone, 5 g/L NaCl) solidified with 0.3% agar. *Proteus mirabilis* minimal salts medium (PMSM) [77] was used for studies requiring defined medium (10.5 g/L K_2_HPO_4_, 4.5 g/L KH_2_PO_4_, 0.47 g/L sodium citrate, 1 g/L (NH_4_)_2_SO_4_, 15 g/L agar, supplemented with 0.001% nicotinic acid, 1mM MgSO_4_, and 0.2% glycerol). Transposon mutants were cultured in LB containing 25 μg/ml kanamycin (Sigma). Additional *P*. *mirabilis* mutants for validation of conditionally essential genes were constructed by insertion of a kanamycin resistance cassette as previously described using the TargeTron system (Sigma) [69]. Resulting mutants were screened by kanamycin selection and PCR (all primers for generation and verification of mutants are provided in S10 Table).

#### Construction of pSAM_AraC

A derivative of pSAM_Ec [78] containing an arabinose-inducible transposase was constructed for generation of the *P*. *mirabilis* transposon mutant library. Briefly, pSAM_Ec was digested with NcoI and EcoRI to excise the transposase, and the fragment was ligated into pBAD_myc_his_a (Invitrogen). The resulting vector was then digested with NdeI and PmeI, creating a fragment containing the transposase, under control of the P_BAD_ promoter, and *araC*. pSAM_Ec was digested with NdeI and AleI to remove P_lac_, and the above fragment was ligated in to generate pSAM_AraC. The construct was verified by restriction digest and PCR, and was propagated in *E*. *coli* S17-1λ*pir*. The final pSAM_AraC construct was deposited in Addgene (plasmid #91569, https://www.addgene.org/91569/).

#### Generation and validation of transposon mutants

A library of random transposon mutants was fashioned by mating a mid-log culture of *E*. *coli* S17-1λ*pir* carrying pSAM_AraC (donor strain) with *P*. *mirabilis* HI4320 (recipient strain) at 4:1 ratio of donor to recipient. Mating mixes were pelleted, incubated at room temperature for 5 min, gently resuspended in 50 μl LB, and incubated at 37°C with aeration for 40 min. Mating mixtures were then spread onto 0.45 μm filter disks (Millipore) on the surface of LB agar plates with 100 μl 1M arabinose (Sigma), and incubated at 30°C for 2 h to induce transposase expression via the P_BAD_ promoter. Filter disks were transferred to LB agar plates with 15 μg/ml tetracycline and 25 μg/ml kanamycin to isolate *P*. *mirabilis* mutants harboring the transposon, mating mixtures were gently transferred from the filters by washing with 100 μl LB, and plates were incubated at 37°C overnight. Input pool freezer stocks were generated by swabbing 10,000 Tet^R^ Kan^R^ colonies into PBS, adjusting to ~2x10^9^ CFU/ml (OD_600_ 2.0), and diluting 1:1 with 50% glycerol for storage at -80°C in 1 ml aliquots.

Randomness of insertions was verified by extracting genomic DNA from individual mutants, digesting with Hin*d*III, and Southern blot with a digoxigenin (Roche)-labeled probe targeting the Kan^R^ cassette within the transposon (S2A Fig). The resulting transposon libraries were tested for frequency of pSAM_AraC retention by plating onto agar with 100 μg/ml ampicillin and by PCR using primers homologous to the vector backbone. Less than 0.001% of mutants retained the pSAM_AraC vector (S2B Fig).

#### Mouse model of CAUTI

Infection studies were carried out as previously described [21, 79, 80] using a modification of the Hagberg protocol [81]. Transposon mutant pools (1 ml volume) were thawed in 9 ml fresh LB with kanamycin and cultured at 37◦C for no more than 10 h. Cultures were then adjusted to an OD_600_ of ~0.2 (2x10^8^ CFU/ml), and diluted 1:100. CBA/J mice (Envigo) were inoculated transurethrally with 50 μl of 2x10^6^ CFU/ml (1x10^5^ CFU/mouse), and a 4 mm segment of sterile silicone tubing (0.64 mm O.D., 0.30 mm I.D., Braintree Scientific, Inc.) was carefully advanced into the bladder during inoculation and retained for the duration of the study as described previously [21, 82–84]. For coinfection experiments, mice were inoculated with 50 μl of a 1:1 mixture of the *P*. *mirabilis* transposon mutant pools and WT *P*. *stuartii* BE2467. For each transposon pool input, 5–10 mice were inoculated for single species infection and 5–10 mice were coinfected with *P*. *stuartii*. Mice were euthanized 4 days post-inoculation (dpi) and bladders, kidneys and spleens were harvested into phosphate-buffered saline (0.128 M NaCl, 0.0027 M KCl, pH 7.4). Notably, catheter segments were not removed from the bladder samples for homogenization, so CFUs for bladder samples represent a catheterized bladder. Tissues were homogenized using an Omni TH homogenizer (Omni International), and a 150 μl aliquot was removed and plated using an Autoplate 4000 spiral plater (Spiral Biotech) for enumeration of colonies using a QCount automated plate counter (Spiral Biotech). The remaining bladder and kidney homogenates were spread plated in their entirety, and colonies were collected, pelleted, and frozen for sequencing. A competitive index (CI) was calculated as follows for all samples in which bacterial burden was above the limit of detection:

$$\text{CI}=\frac{\text{Strain A output}/\text{Strain B output}}{\text{Strain A input}/\text{Strain B input}}$$

Log_10_CI = 0 indicates that the ratio of the strains in the output is similar to the input, and neither strain had an advantage. Log_10_CI>0 indicates that strain A has a competitive advantage over strain B. Log_10_CI<0 indicates that strain B has a competitive advantage over strain A.

#### Illumina sequencing

Genomic DNA was isolated from the five input inocula, with two technical replicates each, and from the bladder and kidney homogenates of each individual mouse (outputs) by hexadecyltrimethyl ammonium bromide (CTAB) precipitation [85]. Samples were enriched for transposon insertion junctions as outlined by Goodman et al. [29]. TapeStation analysis was used to confirm the concentration and purity of the transposon insertion junctions, which were then multiplexed and subjected to V4 single end 50 HiSeq-2500 High-Output sequencing as follows: 1) 5 input samples with 2 replicates each were multiplexed and sequenced on a single lane, 2) *P*. *mirabilis* single-species outputs from 20 mouse bladders were multiplexed and sequenced on one lane, 3) *P*. *mirabilis* single-species outputs from 20 mouse kidneys were multiplexed and sequenced on one lane, 4) *P*. *mirabilis* coinfection with *P*. *stuartii* outputs from 20 mouse bladders were multiplexed and sequenced on two lanes, and 5) *P*. *mirabilis* coinfection with *P*. *stuartii* outputs from 20 mouse kidneys were multiplexed and sequenced on two lanes. Each lane was spiked with 15% bacteriophage φX DNA to overcome low-diversity sequences. Sequencing was performed at the University of Michigan DNA core facility.

#### Mapping of transposon insertion-sites

The chromosome and plasmid sequences of *P*. *mirabilis* HI4320 (NCBI accession numbers NC_010054 and NC_010555) [26] were combined into a single genome sequence with 1000 N’s added between them and the coordinates of genes on NC_010555 adjusted accordingly. The Goodman In-Seq pipeline [29] was applied on the raw reads to perform read filtration, transposon nucleotide removal, debarcoding, alignment, and insertion calling. A script was written to map the insertions onto the *P*. *mirabilis* genome. For each gene, this script used the 80% of the gene body towards the transcriptional start site as the effective gene region, and output the following information to be used for statistical tests: 1) the positions of insertion sites in the effective gene region, 2) the number of insertion sites within the gene region, 3) the dinucleotide bases of each insertion site, and 4) the total number of TA dinucleotides in the effective gene region. The Goodman pipeline includes a cap to only use insertion sites with >3 reads. This cap was removed to allow for statistical modeling of insertion sites with any reads.

#### Estimation of essential genes

Input samples were first corrected for potential read count bias caused by the DNA replication process. Specifically, a mean count in each genomic region was estimated from the LOESS function to obtain a bias factor for each region. The observed insertion counts at any particular location were then divided by the bias factor for their genomic region. A Bayesian mixture model was then used to estimate the rate for insertion counts in each gene assuming the counts follow a Poisson distribution. Absolute essential genes are characterized by a low rate of insertion counts in the input samples, while non-essential genes have a high rate of insertion counts. The rate of insertion counts at non-TA sites (which occurs at a very low level) was estimated for each input sample, allowing for within-sample comparison of the rate for insertion counts. If the insertion rate at the TA sites within a gene was similar to the background insertion rate at non-TA sites within that gene, the gene was classified as an estimated essential gene. JAGS [86] was used to perform the above model and obtain a posterior probability for evaluating the likelihood of each gene being essential. Genes identified as having a 90% probability or greater of being essential were considered “essential genes.” Estimated essential genes were sorted into functional groups using their Clusters of Orthologous Groups of proteins (COG) assignments (https://img.jgi.doe.gov/cgi-bin/edu/main.cgi?section=TaxonDetail&page=cogs&cat=cat&taxon_oid=642555150) [87].

#### Identification of *P*. *mirabilis* fitness factors for single-species and polymicrobial CAUTI

As the urinary tract does not represent a closed system and some loss of transposon mutants may occur due to urination early after inoculation, stringent cutoffs were set to reduce the potential for bias in estimation of fitness factors. First, if a bottleneck was detected for an individual output sample, as indicated by recovery of <100 genes with transposon insertions or insertions at <500 unique sites, the entire sample was excluded from analysis for estimating fitness contribution. Second, the output samples from 20 mice, 4 mice per input pool, were combined for estimation of fitness contribution to a given infection type (single-species vs coinfection) to reduce the potential bias from random loss of mutants due to urination or mouse-to-mouse and pool-to-pool variability. Third, individual genes were only assessed for fitness contribution if the mean of the sum of insertion-site reads was >1000 and the number of insertions in that gene was >5. After applying these cutoffs, the fitness contribution of each gene was estimated in a two-step process. A Confidence distribution (CD) function [88] was first constructed to collect fitness evidence for each insertion by comparing read counts in the input sample to the output samples, with the counts at each insertion-site modelled as a linear function of the condition (input/output). To account for the over-dispersion of the count data produced from the sequencing technique, a precision weight was estimated for each observation from the mean-variance relationship of the log-counts and incorporated into the linear modeling [89]. The CD function was constructed using the slope (log fold-change) estimates (mean, standard error and degrees of freedom) from the linear model. Insertion-level CD functions were then combined to obtain a single CD function for the corresponding gene, and a *P*-value was derived from this combined CD function to infer the fitness effect of that gene [88]. These *P* -values were further adjusted for multiple hypothesis correction [90]. The analysis was performed using a newly-developed R package called Tnseq, which can be installed from the Comprehensive R Archive Network (CRAN). Significant genes for further analysis were selected based on an adjusted *P*-value <0.05 and >2-fold ratio of output over input.

#### Growth curves

Overnight cultures of *P*. *mirabilis* mutants washed 1x in PBS and diluted 1:100 in growth medium. Where indicated, LB and PMSM were supplemented with polymyxin B, l-glutamine, BCAAs, nickel sulfate, or copper sulfate. A Bioscreen-C Automated Growth Curve Analysis System (Growth Curves USA) was utilized to generate growth curves. Cultures were incubated at 37°C with continuous shaking, and OD_600_ readings were taken every 15 min for 16 h. Urine growth curves were performed by diluting overnight cultures as above, inoculating filter-sterilized pooled human urine with a 1:1 mixture of wild-type *P*. *mirabilis* and a mutant, and incubating at 37°C with aeration. Aliquots were taken at the time of inoculation and hourly thereafter for five hours, diluted, and plated onto LB agar with and without kanamycin to determine CFUs of mutant and wild-type. Competitive indices for growth in urine were calculated as described above for murine infection studies.

#### Urease activity

Urease activity of *P*. *mirabilis* mutants was measured as described previously using an existing pool of frozen human urine [21]. Briefly, bacteria were cultured in LB to mid-log phase (OD_600_ = 0.5), centrifuged to pellet, resuspended in filter-sterilized dilute human urine, and incubated at 37°C with aeration. At 15-or 30-minute intervals, 1 ml was removed from each culture, centrifuged to pellet, and resuspended in 1/10 volume of 0.9% saline. Wells of a 96-well plate containing urine, 0.001% WT/vol phenol red, and 250 mM urea were inoculated with 20 μl of the saline resuspension. OD_562_ was measured using a μQuant (BioTek) over a 5-minute kinetic read, and urease activity was expressed as the mean change in optical density per minute (mOD/min), as calculated by the Gen5 software (BioTek).

#### Construction of a vector for allelic exchange in *Providencia stuartii*

An allelic exchange vector for generation of targeted mutants in *P*. *stuartii* BE2467 was created using Phusion high-fidelity DNA polymerase and the NEBuilder HiFi DNA Assembly 2X Master Mix (New England BioLabs) as follows. All primer sequences are provided in S10 Table. First, a silent mutation in pWSK29 [91] was made to ablate the BsaI site located within the β-lactamase (*bla*) cassette coding region. A β-lactamase cassette with flanking BsaI restriction sites was then created from this template using primers AOJ1 and AOJ2, which will be referred to as Fragment 1. Next, three fragments were generated from pRK415 [92]: Fragment 2 contains the *oriV* region from RK2, which was amplified using primers AOJ3 and AOJ4; Fragment 3 contains the *lacZ*α gene and the RK2 origin of transfer (*oriT*), which was amplified using primers AOJ5 and AOJ6; Fragment 4 contains the *tetR* promoter and coding sequence, which was amplified with primers AOJ7 and AOJ8. The *tse2* type 6 secretion system toxin from *Pseudomonas aeruginosa* strain PAO1 was chosen as a negative selection marker for the vector based on the work of Khetrapal et al [93]. Thus, Fragment 5 contains the *tse2* gene, which was amplified from *P*. *aeruginosa* strain PAO1 genomic DNA with primers AOJ9 and AOJ10. Finally, Fragment 6 contains a predicted terminator region from pSIM18 [94] with flanking SapI restriction sites, and was generated using primers AOJ11 and AOJ12. All fragments were gel-purified using the Monarch nucleic acid purification kit (New England BioLabs). Fragments 1–5 were assembled into a circular vector using the NEBuilder HiFi DNA Assembly 2X Master Mix (New England BioLabs), and the resulting vector was transformed into *E*. *coli* EPI300 [95] for propagation. The resulting plasmid and Fragment 6 were then digested with SapI and ligated with T4 DNA ligase (New England BioLabs) to form plasmid pAOJ15, in which expression of the *tse2* toxin is induced by tetracycline or anhydrotetracycline. Assembly of pAOJ15 was verified by restriction digest, and a plasmid map is provided in S6 Fig. The final pAOJ15 construct was deposited in Addgene (plasmid #91567, https://www.addgene.org/91567/).

#### Generation of a *livK* mutant in *Providencia stuartii* strain BE2467

A hygromycin resistance cassette was amplified from pSIM18 [94] using primers AOJ17 and AOJ18. Approximately 1100 bp upstream of the *livK* gene were amplified from *P*. *stuartii* genomic DNA using primers AOJ13 and AOJ14, and approximately 1200 bp downstream of the *livK* gene were amplified using primers AOJ15 and AOJ16. These fragments, along with the hygromycin cassette, were combined into a single fragment using the NEBuilder HiFi DNA Assembly 2X Master Mix, and the entire assembled product was amplified using primers AOJ13 and AOJ16. Adenine overhangs were created for the amplified product using Taq DNA polymerase (New England BioLabs), and the fragment was TOPO cloned into pCR2.1 (ThermoFisher Scientific) and transformed into Top10 *E*. *coli* cells for plasmid propagation. The pCR2.1_*livK*::*hyg* vector and pAOJ15 were digested with KpnI-HF and XbaI (New England BioLabs), ligated together, and transformed into *E*. *coli* S17-1λ*pir*. Assembly of pAOJ15_ *livK*::*hyg* was verified by restriction digest and with primers AOJ19 and AOJ20.

*E*.*coli* S17-1λ*pir* harboring pAOJ15_ *livK*::*hyg* (donor strain) and wild-type *P*. *stuartii* strain BE2467 (recipient strain) were cultured in LB overnight at 37°C with aeration. A sample (250 μl) of each culture was sub-cultured into 25 ml of pre-warmed LB and incubated at 37°C with aeration for 90 min. Cultures were then centrifuged, washed twice with PBS, and combined, incubated at room temperature for 5 min, and gently resuspended in 250 μl of SOC medium (5 g/L yeast extract, 20 g/L tryptone, 0.6 g/L NaCl, 0.2 g/L KCl, 1 g/L MgCl_2_ hexahydrate, 1.2 g/L MgSO_4_ anhydrous, and 20 mM glucose). The cell suspension was then spread onto a 0.45 μm filter disk (Millipore) on the surface of an LB plate and incubated at 37°C for 90 min. The filter was then washed with 2.5 ml SOC and recovered for an additional 90 min at 37°C with aeration. 100 μl aliquots of the recovery culture were spread onto LB agar plates containing 15 μg/ml tetracycline (Sigma), 1 μg/ml anhydrotetracycline (Cayman Chemical), and 150 μg/ml hygromycin B (Goldbio), and incubated overnight at 37°C. Resulting colonies were re-plated onto LB agar containing tetracycline, anhydrotetracycline, and hygromycin B and incubated overnight at 37°C. Isolated colonies were then grown overnight in LB with 150 μg/ml hygromycin B. Genomic DNA was isolated from these cultures using a DNease kit (Qiagen), and verified for the exchange of the majority of the *livK* coding sequence with the hygromycin resistance cassette using primers AOJ19 and AOJ20.

#### Statistical analysis

Significance for growth curves, urease activity, and swarm ring diameter were assessed using two-way analysis of variance (ANOVA). Significant differences in swimming motility were determined by Student’s *t*-test. Wilcoxon signed-rank test was used to determine which mutants were significantly outcompeted by the parental strain in all co-challenge experiments. These analyses were performed using GraphPad Prism, version 7 (GraphPad Software, San Diego, CA). All *P* values are two tailed at a 95% confidence interval.

## Supporting information

S1 Fig Colonization and bottleneck assessment in the CAUTI model. CBA/J mice were transurethrally inoculated with 1x10^5^ CFU of a mixture of a kanamycin-resistant mutant of *P*. *mirabilis* HI4320 and the wild-type strain at the following ratios: 1:1, 1:100, and 1:1000. In all cases, a 4 mm segment of sterile silicone catheter tubing was carefully advanced into the bladder during inoculation and retained for the duration of the study. (A and B) Urine was collected and mice were euthanized either 4-h or 4-days post-inoculation, and the bladder and kidneys were homogenized and plated onto LB agar with and without kanamycin to determine bacterial burden. Solid lines represent the median, and dashed lines indicate the limit of detection. (C) A competitive index (CI) was calculated for the kanamycin-resistant mutant ratio using the ratio of mutant to wild-type in each organ divided by the ratio of mutant to wild-type from the inoculum. Dashed lines indicate a competitive index of 1, or a 1:1 ratio of mutant to wild-type indicative of lack of a bottleneck in the CAUTI model.
(TIF)

S2 Fig *P*. *mirabilis* transposon mutant libraries contain random insertions. (A) Genomic DNA was extracted from *P*. *mirabilis* transposon mutants and digested with HindIII. Southern blotting was performed using a dioxigenin probe against the kanamycin-resistance cassette contained within the transposon. The varying fragment sizes are indicative of transposon insertion into a variety of locations within the genome, and the presence of a single band within a given lane indicates that a single transposon insertion event occurred, while double bands may indicate incomplete digest or insertion at two locations. Seven out of 55 colonies tested had double bands. (B) Representative colony PCR of kanamycin-resistant *P*. *mirabilis* transposon mutants using primers homologous to the Kan^R^ cassette (top) and the vector backbone (bottom) to verify that transposon mutants lost the vector backbone. Only mutants with a band corresponding to the backbone primers retained ampicillin resistance from the backbone vector. Of 10^5^ mutants tested for growth on ampicillin, only 0.001% retained the pSAM_AraC vector.
(TIF)

S3 Fig Chromosomal distribution of *P*. *mirabilis* genes required for bladder and kidney colonization during single-species CAUTI. Chromosomal location of all genes identified as candidate fitness factors for bladder or kidney colonization during single-species CAUTI. Arrows indicate open reading frames of the *P*. *mirabilis* chromosome (black) or plasmid pHI4320 (pink), with arrow heads indicating direction of transcription. Each color-block on the gray line represents a gene that was identified as a significant fitness factor for bladder colonization (red), kidney colonization (green), or both (blue).
(TIF)

S4 Fig *In vitro* co-challenge of *ilvD* during growth in human urine. Filter-sterilized pooled human urine from healthy donors was inoculated with a 1:1 mixture of wild-type *P*. *mirabilis* and *ilvD* (A and B), or a 1:1 mixture of this combination and wild-type *P*. *stuartii* (C and D). Cultures were incubated at 37°C for 5 hours, and sampled hourly for determination of CFUs (A and C). Error bars represent mean and error for three independent replicates. No differences in growth were detected by two-way ANOVA with post-hoc test for significance. A competitive index was calculated for *ilvD* at each hourly timepoint (B and D), and no significant fitness defects or advantages were detected by the Wilxocon signed rank test.
(TIF)

S5 Fig Growth of wild-type *P*. *stuartii* and the *livK* mutant in minimal medium. Growth of *P*. *stuartii* BE2467 and the *livK* mutant was measured in PMSM minimal medium (A) or PMSM supplemented with 50μM copper sulfate to inhibit BCAA biosynthesis (B). Strains were cultured in medium alone (black/gray), supplemented with 0.5 mM leucine (blue), or supplemented with 0.5 mM total concentration of all three BCAAs (orange). Graphs are representative of three independent experiments. Error bars represent mean ± SD from three technical replicates. The difference in growth between the *livK* mutant and wild-type when supplemented with 0.5 mM leucine in panel B was significant by two-way ANOVA (*P*<0.0019).
(TIF)

S6 Fig Plasmid map of pAOJ15, a vector for allelic exchange in *Providencia stuartii*. An allelic exchange vector for generation of targeted mutants in *P*. *stuartii* BE2467 was created using NEBuilder HiFi DNA Assembly 2X Master Mix and the following constructs: a β-lactamase cassette with flanking BsaI restriction sites, the *oriV*, *lacZ*α, and *oriT* from RK2, the *tetR* promoter and coding sequence, the *tse2* type 6 secretion system toxin from *Pseudomonas aeruginosa* strain PAO1, and a predicted terminator region from pSIM18. This vector can be used for allelic exchange by inserting a DNA fragment, with or without an antibiotic resistance cassette for selection, containing ~1100 bp of the flanking regions of the gene to be disrupted into the multiple cloning site. This construct has been deposited in Addgene (plasmid #91567, https://www.addgene.org/91567/).
(TIF)

S1 Table Genes essential for growth of *P*. *mirabilis* in rich medium. (XLSX)

S2 Table *P*. *mirabilis* fitness factors specific for colonization of the catheterized bladder during single-species infection. (XLSX)

S3 Table *P*. *mirabilis* fitness factors specific for colonization of the kidneys during single-species infection. (XLSX)

S4 Table *P*. *mirabilis* fitness factors for colonization of both the catheterized bladder and kidneys during single-species infection. (XLSX)

S5 Table *P*. *mirabilis* fitness factors specific for colonization of the catheterized bladder during polymicrobial infection. (XLSX)

S6 Table *P*. *mirabilis* fitness factors specific for colonization of the kidneys during polymicrobial infection. (XLSX)

S7 Table *P*. *mirabilis* fitness factors for colonization of both the catheterized bladder and kidneys during polymicrobial infection. (XLSX)

S8 Table *P*. *mirabilis* genes for which transposon insertions provided a fitness defect during single-species infection but a potential advantage during polymicrobial infection. (XLSX)

S9 Table *P*. *mirabilis* genes involved in synthesis and transport of branched chain amino acids. (XLSX)

S10 Table Primers used in this study. (DOCX)
