# Supplementary figures and images for: Genome-wide transposon mutagenesis of Proteus mirabilis: Essential genes, fitness factors for catheter-associated urinary tract infection, and the impact of polymicrobial infection on fitness requirements
Source: PLoS Pathog. 2017 Jun 14;13(6):e1006434. doi: 10.1371/journal.ppat.1006434 (PMC5484520; doi:10.1371/journal.ppat.1006434)

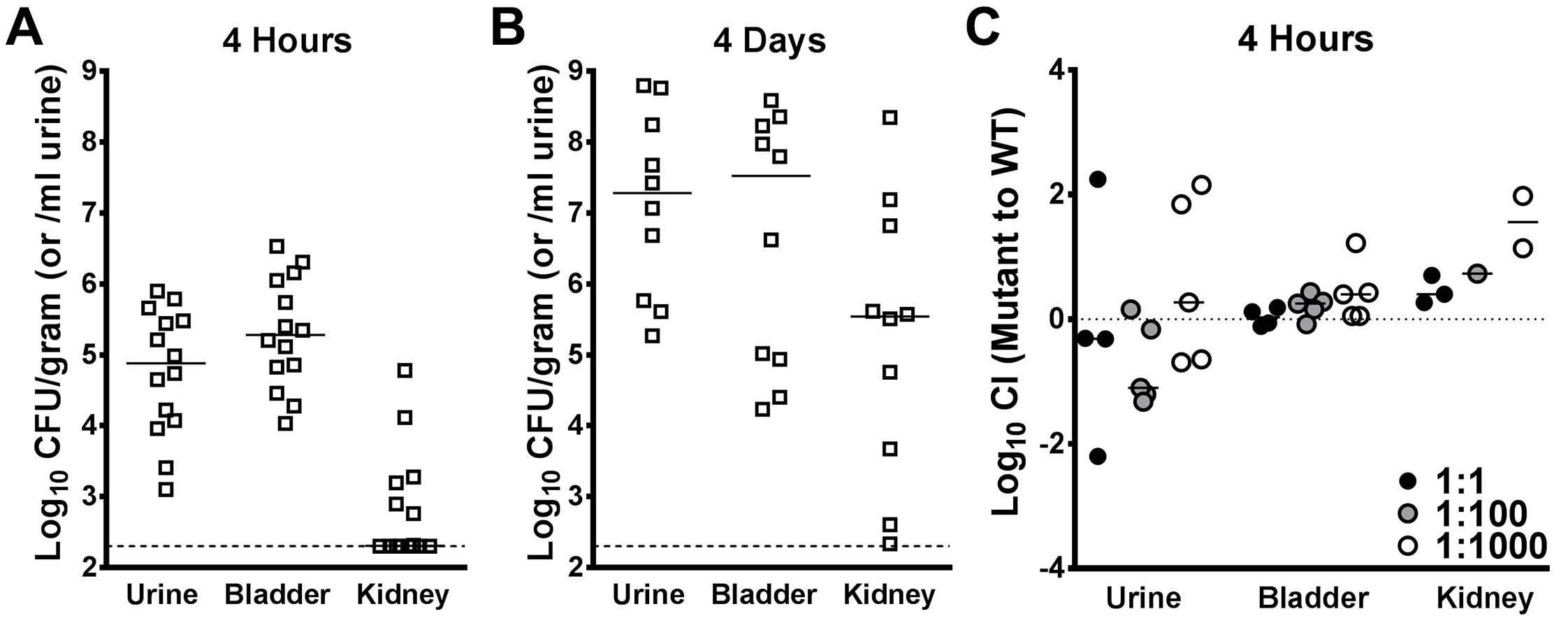

Supplement: S1 Fig — CBA/J mice were transurethrally inoculated with 1x10^5^ CFU of a mixture of a kanamycin-resistant mutant of P. mirabilis HI4320 and the wild-type strain at the following ratios: 1:1, 1:100, and 1:1000. In all cases, a 4 mm segment of sterile silicone catheter tubing was carefully advanced into the bladder during inoculation and retained for the duration of the study. (A and B) Urine was collected and mice were euthanized either 4-h or 4-days post-inoculation, and the bladder and kidneys were homogenized and plated onto LB agar with and without kanamycin to determine bacterial burden. Solid lines represent the median, and dashed lines indicate the limit of detection. (C) A competitive index (CI) was calculated for the kanamycin-resistant mutant ratio using the ratio of mutant to wild-type in each organ divided by the ratio of mutant to wild-type from the inoculum. Dashed lines indicate a competitive index of 1, or a 1:1 ratio of mutant to wild-type indicative of lack of a bottleneck in the CAUTI model. (TIF) [file ppat.1006434.s001.tif]

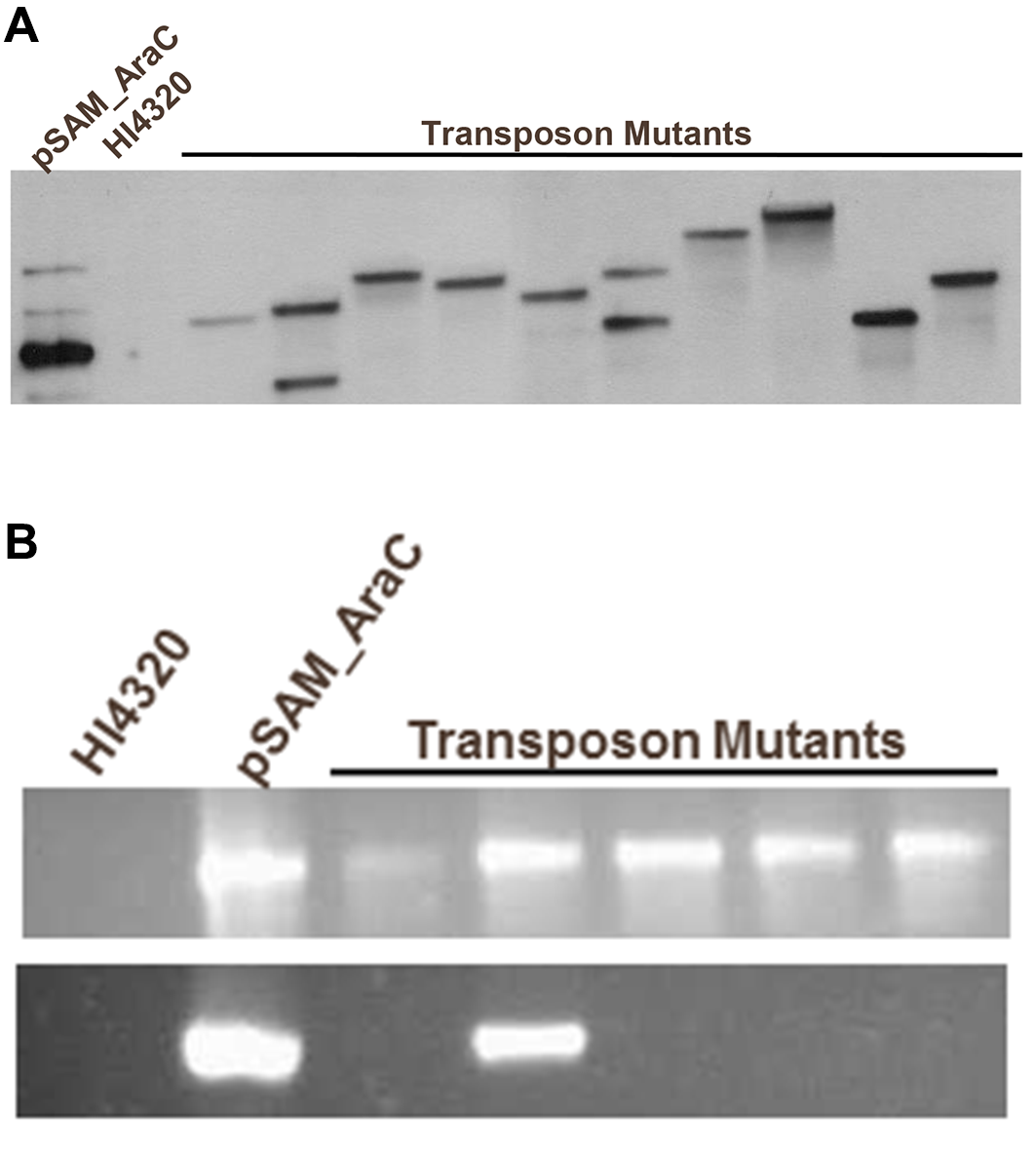

Supplement: S2 Fig — (A) Genomic DNA was extracted from P. mirabilis transposon mutants and digested with HindIII. Southern blotting was performed using a dioxigenin probe against the kanamycin-resistance cassette contained within the transposon. The varying fragment sizes are indicative of transposon insertion into a variety of locations within the genome, and the presence of a single band within a given lane indicates that a single transposon insertion event occurred, while double bands may indicate incomplete digest or insertion at two locations. Seven out of 55 colonies tested had double bands. (B) Representative colony PCR of kanamycin-resistant P. mirabilis transposon mutants using primers homologous to the KanR cassette (top) and the vector backbone (bottom) to verify that transposon mutants lost the vector backbone. Only mutants with a band corresponding to the backbone primers retained ampicillin resistance from the backbone vector. Of 105 mutants tested for growth on ampicillin, only 0.001% retained the pSAM_AraC vector. (TIF) [file ppat.1006434.s002.tif]

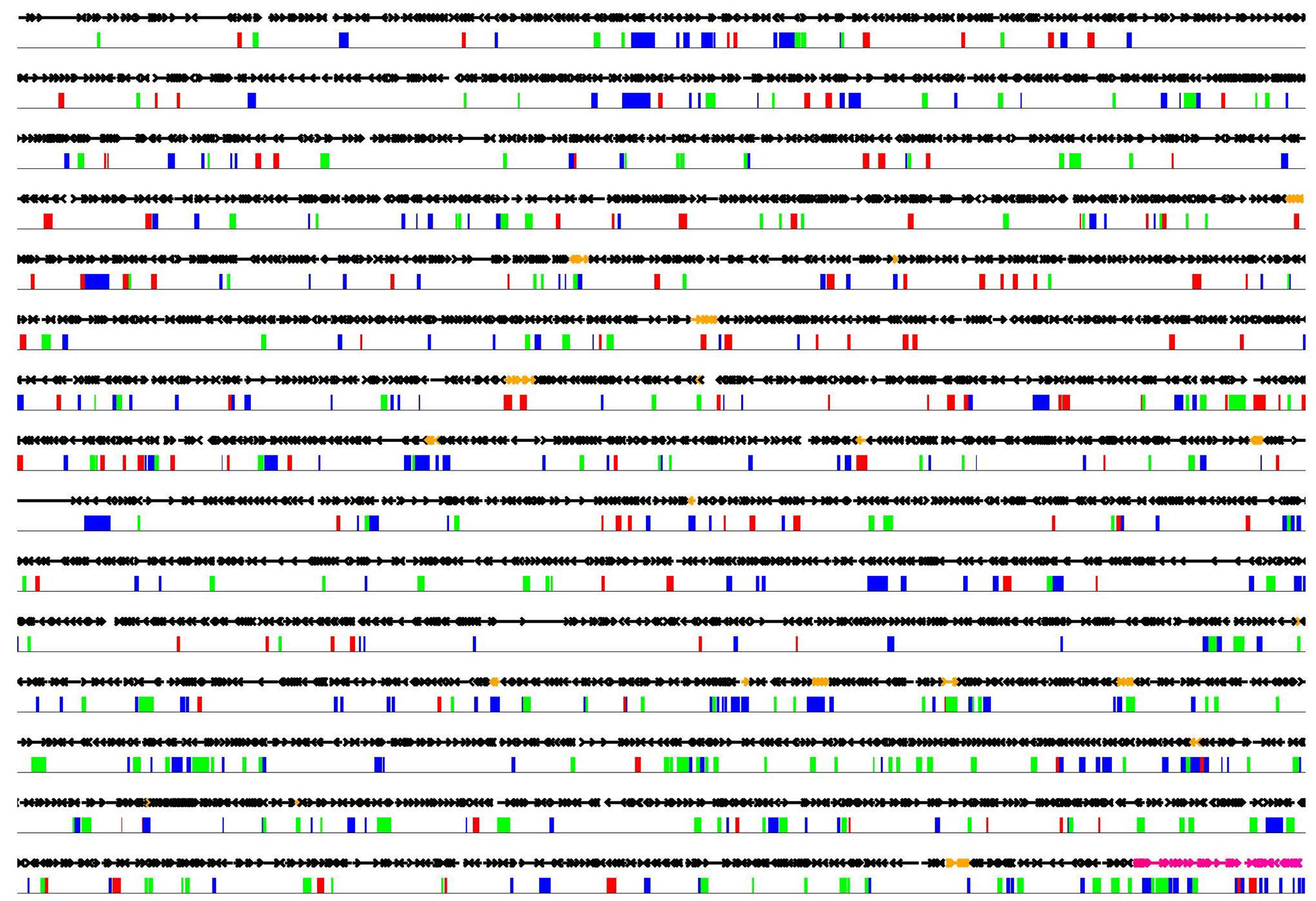

Supplement: S3 Fig — Chromosomal location of all genes identified as candidate fitness factors for bladder or kidney colonization during single-species CAUTI. Arrows indicate open reading frames of the P. mirabilis chromosome (black) or plasmid pHI4320 (pink), with arrow heads indicating direction of transcription. Each color-block on the gray line represents a gene that was identified as a significant fitness factor for bladder colonization (red), kidney colonization (green), or both (blue). (TIF) [file ppat.1006434.s003.tif]

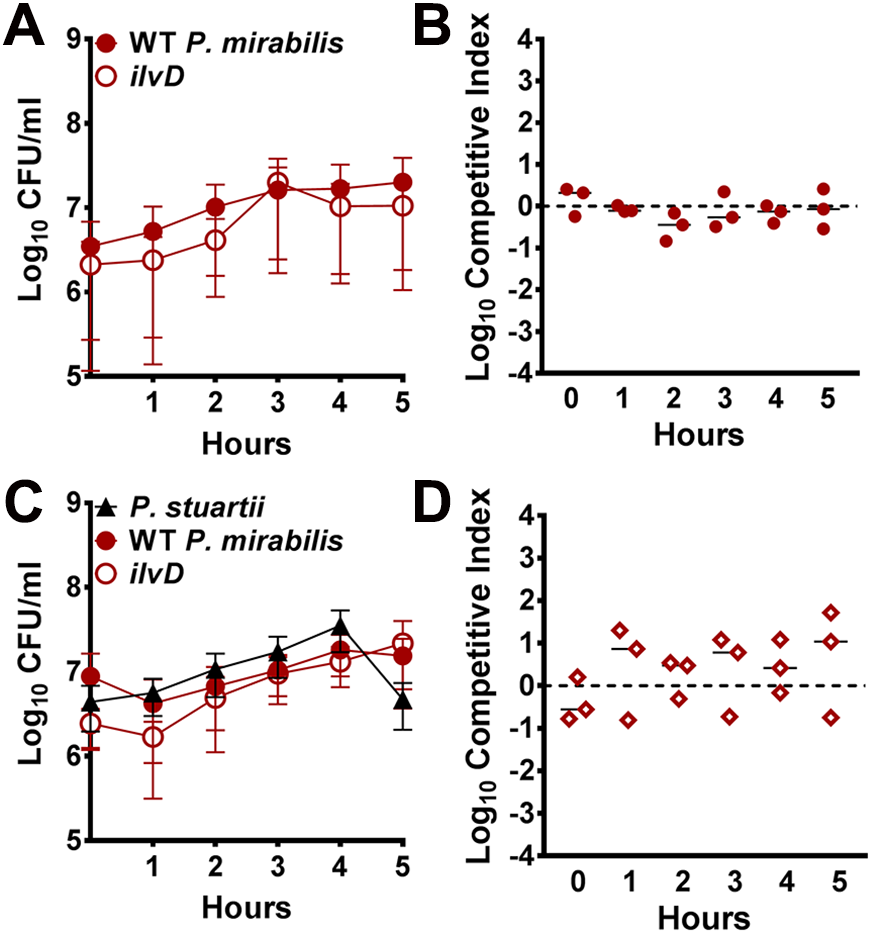

Supplement: S4 Fig — Filter-sterilized pooled human urine from healthy donors was inoculated with a 1:1 mixture of wild-type P. mirabilis and ilvD (A and B), or a 1:1 mixture of this combination and wild-type P. stuartii (C and D). Cultures were incubated at 37°C for 5 hours, and sampled hourly for determination of CFUs (A and C). Error bars represent mean and error for three independent replicates. No differences in growth were detected by two-way ANOVA with post-hoc test for significance. A competitive index was calculated for ilvD at each hourly timepoint (B and D), and no significant fitness defects or advantages were detected by the Wilxocon signed rank test. (TIF) [file ppat.1006434.s004.tif]

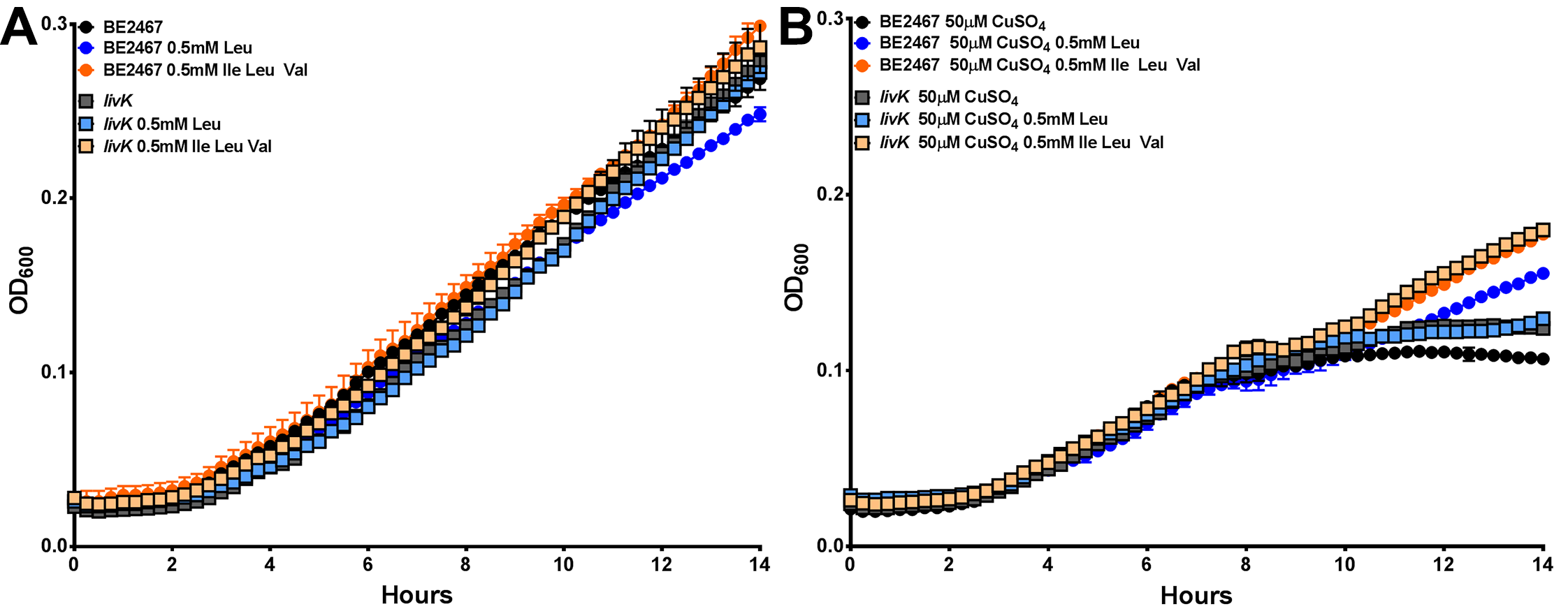

Supplement: S5 Fig — Growth of P. stuartii BE2467 and the livK mutant was measured in PMSM minimal medium (A) or PMSM supplemented with 50μM copper sulfate to inhibit BCAA biosynthesis (B). Strains were cultured in medium alone (black/gray), supplemented with 0.5 mM leucine (blue), or supplemented with 0.5 mM total concentration of all three BCAAs (orange). Graphs are representative of three independent experiments. Error bars represent mean ± SD from three technical replicates. The difference in growth between the livK mutant and wild-type when supplemented with 0.5 mM leucine in panel B was significant by two-way ANOVA (P<0.0019). (TIF) [file ppat.1006434.s005.tif]

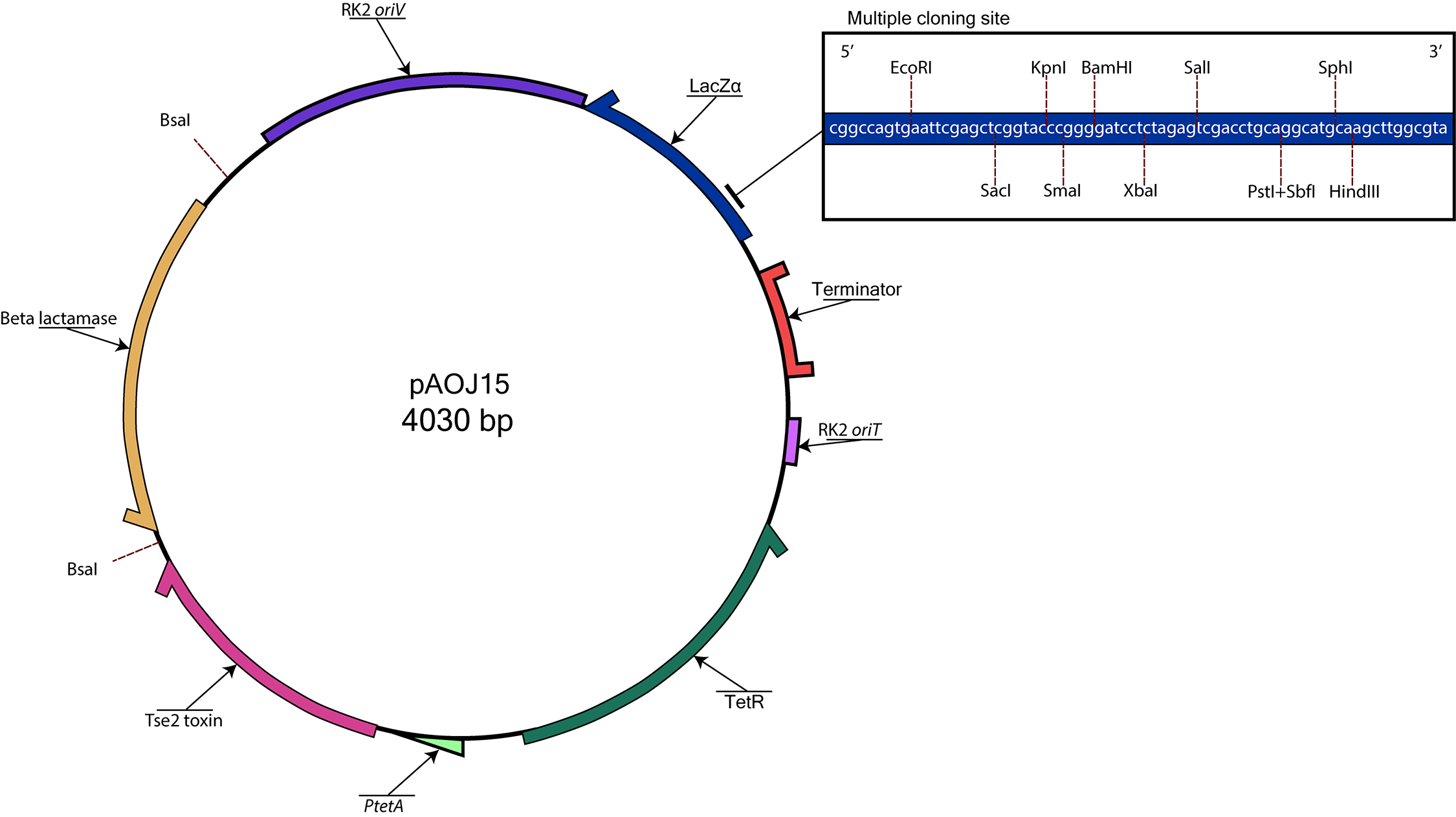

Supplement: S6 Fig — An allelic exchange vector for generation of targeted mutants in P. stuartii BE2467 was created using NEBuilder HiFi DNA Assembly 2X Master Mix and the following constructs: a β-lactamase cassette with flanking BsaI restriction sites, the oriV, lacZα, and oriT from RK2, the tetR promoter and coding sequence, the tse2 type 6 secretion system toxin from Pseudomonas aeruginosa strain PAO1, and a predicted terminator region from pSIM18. This vector can be used for allelic exchange by inserting a DNA fragment, with or without an antibiotic resistance cassette for selection, containing ~1100 bp of the flanking regions of the gene to be disrupted into the multiple cloning site. This construct has been deposited in Addgene (plasmid #91567, https://www.addgene.org/91567/). (TIF) [file ppat.1006434.s006.tif]
