# Supplementary material for: Genome-wide transposon mutagenesis of Proteus mirabilis: Essential genes, fitness factors for catheter-associated urinary tract infection, and the impact of polymicrobial infection on fitness requirements
Source: PLoS Pathog. 2017 Jun 14;13(6):e1006434. doi: 10.1371/journal.ppat.1006434 (PMC5484520; doi:10.1371/journal.ppat.1006434)
Supplement: S10 Table — (DOCX) [file ppat.1006434.s016.docx]

| **Name** | | | **Sequence (5’ – 3’)** | | | | | | **Purpose** |
| --- | --- | --- | --- | --- | --- | --- | --- | --- | --- |
| ***Primers for generation and verification of the P. mirabilis transposon mutant library*** | | | | | | | | | |
| **KanFSB** | | | | ACAAGATGGATTGCACGCAG | Forward primer for amplifying kanamycin resistance cassette for Southern blot probe. | | | | |
| **KanRSB** | | | | CTGATGCTCTTCGTCCAGAT | Reverse primer for amplifying kanamycin resistance cassette for Southern blot probe. | | | | |
| **TnVer** | | | | ACAGGTTGGATGATAAGTCC | Reverse primer for verification of transposon insertion into *P. mirabilis* HI4320. | | | | |
| **KanRFor** | | | | AAGCAGGTAGCTTGCAGTGG | Forward primer for verification of transposon insertion into *P. mirabilis* HI4320. | | | | |
| **MluIAp5’** | | | | ACCAATGCTTAATCAGTGAGG | Forward primer homologous to pSAM vector backbone. | | | | |
| **MluIAp3’** | | | | ACGAAAGGGCCTCGTGATAC | Reverse primer homologous to pSAM vector backbone. | | | | |
| ***TargeTron primers for disrupting P. mirabilis genes*** | | | | | | | | | |
| ***pldA*IBS1** | AAAAAAGCTTATAATTATCCTTAAAATTCCAGATAGTGCGCCCAGATAGGGTG | | | | | | | Insertion of a kanamycin resistance cassette into *pldA* | |
| ***pldA*EBS1** | CAGATTGTACAAATGTGGTGATAACAGATAAGTCCAGATAAGTAACTTACCTTTCTTTGT | | | | | | |  |  |
| ***pldA*EBS2** | TGAACGCAAGTTTCTAATTTCGGTTAATTTCCGATAGAGGAAAGTGTCT | | | | | | |  |  |
| **1518IBS1** | AAAAAAGCTTATAATTATCCTTAATTAACAAATAGGTGCGCCCAGATAGGGTG | | | | | | | Insertion of a kanamycin resistance cassette into PMI1518 | |
| **1518EBS1** | CAGATTGTACAAATGTGGTGATAACAGATAAGTCAAATAGCTAAACTTACCTTTCTTTGT | | | | | | |  |  |
| **1518EBS2** | TGAACGCAAGTTTCTAATTTCGGTTTTAATCCGATAGAGGAAAGTGTCT | | | | | | |  |  |
| ***argR*IBS1** | AAAAAAGCTTATAATTATCCTTACATAACCACTCAGTGCGCCCAGATAGGGTG | | | | | | | Insertion of a kanamycin resistance cassette into *argR* | |
| ***argR*EBS1** | CAGATTGTACAAATGTGGTGATAACAGATAAGTCCACTCAGTTAACTTACCTTTCTTTGT | | | | | | |  |  |
| ***argR*EBS2** | TGAACGCAAGTTTCTAATTTCGGTTTTATGTCGATAGAGGAAAGTGTCT | | | | | | |  |  |
| ***ilvD*IBS1** | AAAAAAGCTTATAATTATCCTTACATTGCGCAGATGTGCGCCCAGATAGGGTG | | | | | | | Insertion of a kanamycin resistance cassette into *ilvD* | |
| ***ilvD*EBS1** | CAGATTGTACAAATGTGGTGATAACAGATAAGTCGCAGATGCTAACTTACCTTTCTTTGT | | | | | | |  |  |
| ***ilvD*EBS2** | TGAACGCAAGTTTCTAATTTCGGTTCAATGTCGATAGAGGAAAGTGTCT | | | | | | |  |  |
| ***lon*IBS1** | AAAAAAGCTTATAATTATCCTTACGTGACCAAGAGGTGCGCCCAGATAGGGTG | | | | | | | Insertion of a kanamycin resistance cassette into *lon* | |
| ***lon*EBS1** | CAGATTGTACAAATGTGGTGATAACAGATAAGTCCAAGAGGTTAACTTACCTTTCTTTGT | | | | | | |  |  |
| ***lon*EBS2** | TGAACGCAAGTTTCTAATTTCGGTTTCACGTCGATAGAGGAAAGTGTCT | | | | | | |  |  |
| ***Verification primers for P. mirabilis TargeTron mutants*** | | | | | | | | | |
| ***pldA_F*** | | AGAGGCGATTGCCAGTTATG | | | | Verification of disruption of *pldA* with a kanamycin resistance cassette | | | |
| ***pldA_R*** | | CCGACACCGATACGAGTTTG | | | |  |  |  |  |
| **1518_F** | | AAGTGCGTGGTTAAGTGTCC | | | | Verification of disruption of PMI1518 with a kanamycin resistance cassette | | | |
| **1518_R** | | GCCGACCAATTCCCTTGTAA | | | |  |  |  |  |
| ***argR_*F** | | TCACAAGGCGAGATTGTAACTG | | | | Verification of disruption of *argR* with a kanamycin resistance cassette | | | |
| ***argR_*R** | | GATACTTCCGAGGATCCCTTCT | | | |  |  |  |  |
| ***ilvD_*F** | | GGGTGGTGTGGCTAAAGAAT | | | | Verification of disruption of *ilvD* with a kanamycin resistance cassette | | | |
| ***ilvD_*R** | | CCATTACCCGGTTGCGATAA | | | |  |  |  |  |
| ***lon_*F** | | TGAAACTGCCTGACGGAAC | | | | Verification of disruption of *lon* with a kanamycin resistance cassette | | | |
| ***lon_*R** | | TCGCATGCAATGAGGCTAATA | | | |  |  |  |  |
| ***Primers for generating Providencia stuartii allelic exchange vector*** | | | | | | | | | |
| **AOJ1** | | GAACCATGAGACCGCTCAGTGGAACGAAAACTC | | | | | Generation of Fragment 1 (beta-lactamase from modified pWSK29) | | |
| **AOJ2** | | CAAAAATGGCAGGAGACCGCTTACAATTTAGGTGGCA | | | | |  |  |  |
| **AOJ3** | | CTAAATTGTAAGCGGTCTCCTGCCATTTTTGGGGTGAG | | | | | Generation of Fragment 2 (*oriV* from RK2) | | |
| **AOJ4** | | AGTTGGATACCTCGCGGAAAACTTG | | | | |  |  |  |
| **AOJ5** | | CAAGTTTTCCGCGAGGTATCCAACTGTTGGGAAGGGCG | | | | | Generation of Fragment 3 (*oriT* + l*acZ*α from RK2) | | |
| **AOJ6** | | CTTTTTCATGCCCTTCGTGTAGACTTTCCTTGGTG | | | | |  |  |  |
| **AOJ7** | | AGTCTACACGAAGGGCATGAAAAAGCCCGTA | | | | | Generation of Fragment 4 (*tetR* + P*tetA* from RK2) | | |
| **AOJ8** | | CCCTGTGGATTGAACGCGCGGATTCTT | | | | |  |  |  |
| **AOJ9** | | CGCGTTCAATCCACAGGGAAGTCCACGCAT | | | | | Generation of Fragment 5 (*tse2* from *P. aeruginosa* PAO1) | | |
| **AOJ10** | | CTGAGCGGTCTCATGGTTCGGTCTGGGGTTTCAGGTTCAT | | | | |  |  |  |
| **AOJ11** | | GCTCTTCTTGAGTCAGACCCCGTAGAAAAGA | | | | | Generation of Fragment 6 (terminator region from pSIM18 with SapI sites) | | |
| **AOJ12** | | AAAAAGAACGAAGAGCCGATCGTGAGGCAAAGAAAA | | | | |  |  |  |
| **AOJ13** | | AAACGACGGCCAGTGAACCACAAGCTTCAGGGTAA | | | | | Amplification of ~1100 bp upstream of *P. stuartii livK* | | |
| **AOJ14** | | CTTTGCCTCACGATCATTCTGTCGTCCCCATCTTC | | | | |  |  |  |
| **AOJ15** | | TCAATTGTTATCAGCTTACGTCAAGCGAAGCAATC | | | | | Amplification of ~1200 bp downstream of *P. stuartii livK* | | |
| **AOJ16** | | TACCGAGCTCGAATTCCAAGTAGTACTCCCGCTAC | | | | |  |  |  |
| **AOJ17** | | GATCGTGAGGCAAAGAAAAC | | | | | Amplification of the hygromycin resistance cassette | | |
| **AOJ18** | | GCTGATAACAATTGAGCAAGA | | | | |  |  |  |
| ***Primers for verifying disruption of livK in P. stuartii BE2467*** | | | | | | | | | |
| **AOJ19** | | CAAACACAAACATGCAGAACC | | | | | Verification of hygromycin resistance cassette and *livK* upstream and downstream flanking regions in pAOJ15_*livK::hyg* | | |
| **AOJ20** | | ACCAAATCTTCTGGCTCTCG | | | | |  |  |  |
